# Composite Measures of Physical Fitness to Discriminate Between Healthy Aging and Heart Failure: The COmPLETE Study

**DOI:** 10.3389/fphys.2020.596240

**Published:** 2020-12-15

**Authors:** Jonathan Wagner, Raphael Knaier, Karsten Königstein, Christopher Klenk, Justin Carrard, Eric Lichtenstein, Hubert Scharnagl, Winfried März, Henner Hanssen, Timo Hinrichs, Arno Schmidt-Trucksäss, Konstantin Arbeev

**Affiliations:** ^1^Department of Sport, Exercise and Health, Faculty of Medicine, University of Basel, Basel, Switzerland; ^2^Clinical Institute of Medical and Chemical Laboratory Diagnostics, Medical University of Graz, Graz, Austria; ^3^Medical Clinic V, Medical Faculty Mannheim, University of Heidelberg, Mannheim, Germany; ^4^SYNLAB Academy, SYNLAB Holding Deutschland GmbH, Mannheim, Germany; ^5^Biodemography of Aging Research Unit, Social Science Research Institute, Duke University, Durham, NC, United States

**Keywords:** fitness, aging, heart failure, statistical distance, cardiopulmonary exercise test, cardiorespiratory fitness, strength, physiological dysregulation

## Abstract

**Background:**

Aging and changing age demographics represent critical problems of our time. Physiological functions decline with age, often ending in a systemic process that contributes to numerous impairments and age-related diseases including heart failure (HF). We aimed to analyze whether differences in composite measures of physiological function [health distance (HD)], specifically physical fitness, between healthy individuals and patients with HF, can be observed.

**Methods:**

The COmPLETE Project is a cross-sectional study of 526 healthy participants aged 20–91 years and 79 patients with stable HF. Fifty-nine biomarkers characterizing fitness (cardiovascular endurance, muscle strength, and neuromuscular coordination) and general health were assessed. We computed HDs as the Mahalanobis distance for vectors of biomarkers (all and domain-specific subsets) that quantified deviations of individuals’ biomarker profiles from “optimums” in the “reference population” (healthy participants aged <40 years). We fitted linear regressions with HD outcomes and disease status (HF/Healthy) and relevant covariates as predictors and logistic regressions for the disease outcome and sex, age, and age^2^ as covariates in the base model and the same covariates plus combinations of one or two HDs.

**Results:**

Nine out of 10 calculated HDs showed evidence for group differences between Healthy and HF (*p* ≤ 0.002) and most models presented a negative estimate of the interaction term age by group (*p* < 0.05 for eight HDs). The predictive performance of the base model for HF cases significantly increased by adding HD *General health* or HD *Fitness* [areas under the receiver operating characteristic (ROC) curve (AUCs) 0.63, 0.89, and 0.84, respectively]. HD *Cardiovascular endurance* alone reached an AUC of 0.88. Further, there is evidence that the combination of HDs *Cardiovascular endurance* and *General health* shows superior predictive power compared to single HDs.

**Conclusion:**

HD composed of physical fitness biomarkers differed between healthy individuals and patients with HF, and differences between groups diminished with increasing age. HDs can successfully predict HF cases, and HD *Cardiovascular endurance* can significantly increase the predictive power beyond classic clinical biomarkers. Applications of HD could strengthen a comprehensive assessment of physical fitness and may present an optimal target for interventions to slow the decline of physical fitness with aging and, therefore, to increase health span.

## Introduction

Aging and changing age demographics potentially represent one of the most critical problems of our time (Petsko, 2008; Olshansky et al., 2009). The shift of the major causes of morbidity toward chronic disease, coupled with changing age demographics, likely leads to an epidemic of age-driven chronic disease (Seals et al., 2016). Cardiovascular disease is the leading cause of death worldwide (Mendis et al., 2015). It includes heart failure (HF) which is a complex multisystem clinical syndrome. The prevalence of HF continues to rise in sync with the aging population (Ponikowski et al., 2014). Physiological functions decline with age, and these declines often end in a systemic process that contributes to numerous physiological impairments and age-related diseases, including HF (Cai and Harrison, 2000; Seals et al., 2014). The ability to perform physical tasks is critical for maintaining overall functional capacity. Physical fitness is one domain of physiological functions declining with advancing age (Cooper et al., 2011; Studenski et al., 2011; Reid and Fielding, 2012). Physical fitness measures are biomarkers of health, predicting quality of life, disability, and mortality (Fried and Guralnik, 1997; Rantanen et al., 1999; Manini et al., 2007; Studenski et al., 2011). The inverse relationship between cardiovascular risk factors or cardiovascular disease and physical fitness markers such as cardiorespiratory fitness or hand grip strength have been widely described (Ross et al., 2016; Celis-Morales et al., 2018). Physical fitness markers can be separated into three subdomains: cardiovascular endurance, muscle strength, and neuromuscular coordination (Caspersen et al., 1985). So far, a narrow focus on non-physical biomarkers in clinical assessments, however, persists. Further, when physical function is assessed, it is often performed only by a single parameter such as the measurement of grip strength, gait speed, or some measure of cardiorespiratory fitness. In addition, available physical performance batteries are not suitable to provide essential information on all physical fitness components across a wide age spectrum from 20 to 90 years of age. Physical fitness including all components has not been studied comprehensively so far (Wagner et al., 2019).

Measurements of different physiological biomarkers, particularly physical fitness ones, provide an opportunity for personalized predictions of upcoming changes in an individual’s health and onset of diseases and, eventually, death (Seals et al., 2016). Such biomarkers can manifest underlying age-related changes in physiological dysregulation that propagate to deteriorating health and result in increased risks of adverse outcomes with age. Both individual biomarkers and composite measures based on multiple biomarkers have been studied in relation to morbidity and mortality outcomes (see, e.g., recent reviews in Arbeev et al., 2016; Mitnitski and Rockwood, 2019). Recently, the statistical (Mahalanobis) distance (D_M_; denoted in the context of this paper as health distance, HD; we use D_M_ in this paragraph in discussion of previous literature and HD in further narrative), constructed based on the joint distribution of multiple biomarkers, was proposed as a composite measure that can represent the level of physiological dysregulation in an aging organism (Cohen et al., 2013; Arbeev et al., 2019, 2020a). It can be used as a measure of aging-related declines in robustness and resilience and as a preclinical indicator of an individual’s transition from a healthy to an unhealthy state (Arbeev et al., 2019). Numerous studies have confirmed the association of D_M_ with mortality, diseases, and aging-related outcomes (Cohen et al., 2013, 2015; Milot et al., 2014; Arbeev et al., 2019, 2020a), and there is emerging evidence on genetic determinants of the rates of physiological dysregulation represented by D_M_ (Arbeev et al., 2020b). However, applications of this measure to broader sets of biomarkers, including physical fitness ones, and studies of their association with impaired health status such as HF are still lacking. In this paper, we constructed HD using, for the first time, biomarkers of physical fitness to test whether the levels of HD are associated with health status in the COmPLETE project (Wagner et al., 2019). Biomarkers of all physical fitness domains were included.

The aims of this study were to: (1) compare composite measures (HD) between healthy individuals and patients with HF, (2) describe how HD changes with increasing age in health and HF, (3) compare domains of physical fitness summarized in multiple HDs against each other and against HD of standard clinical biomarkers, and (4) analyze whether HD can increase sensitivity and specificity in the discrimination process between healthy individuals and patients with HF.

## Materials and Methods

### Population and Recruitment

The COmPLETE project is a cross-sectional single-center study performed between 2018 and 2019 in Basel, Switzerland. The project comprised two parts, COmPLETE-Health and COmPLETE-Heart. COmPLETE-Health included 526 healthy men and women aged 20–91 years equally distributed across age decades and sex. The participants had a body mass index <30 kg/m^2^, and were non-smokers or ex-smokers for more than 10 years. Exclusion criteria included any kind of exercise-limiting chronic disease and blood pressure > 160/100 mmHg. COmPLETE-Heart included 79 cardiac patients with stable HF with New York Heart Association (NYHA) functional classes I–III; thus, symptoms and signs have remained unchanged for at least 1 month. Diagnosis of HF was confirmed on clinical history, physical examination, assessment of N-terminal pro Brain Natriuretic Peptide (NT-proBNP), and echocardiographically demonstrated relevant structural heart disease or diastolic dysfunction according to the European Society of Cardiology guidelines (Ponikowski et al., 2016). The exact recruitment procedure and the full list of inclusion and exclusion criteria can be found in the study protocol (Wagner et al., 2019).

### Setting

This study was carried out at the Department of Sport, Exercise, and Health at the University of Basel, Switzerland, and was funded by the Swiss National Science Foundation (Grant No. 182815). It was approved by the Ethics Committee of Northwestern and Central Switzerland (EKNZ 2017-01451). Written informed consent was obtained from all study participants prior to inclusion.

### Data

#### General Health Domain

Height and body mass were measured to the nearest 0.5 cm and 0.1 kg, respectively, and the body mass index was calculated. A four-segment bioelectrical impedance analysis was conducted (Inbody 720; Inbody Co. Ltd., Seoul, South Korea) to measure percentage body fat, lean body mass, and skeletal muscle mass. Resting systolic and diastolic blood pressures, resting heart rate, pre-ejection period, ejection time of the left ventricle, brachial-ankle pulse wave velocity (baPWV), and cardio–ankle vascular index (CAVI) were measured with the participant in the supine position using a non-invasive vascular screening system (VaSera VS-1500 N; Fukuda Denshi, Tokyo, Japan). Smoking status was assessed by telephone interview prior to the appointment, whereas physicians reviewed medical history and medications by onsite questionnaires. Forced vital capacity (FVC) and forced expiratory volume in one second (FEV_1_), objective parameters of respiratory function, were measured in accordance with the American Thoracic Society/European Respiratory Society guidelines (Miller et al., 2005) immediately before the exercise test (Balady et al., 2010). Blood samples were drawn via venipuncture by trained medical staff in fasting status (at least 3 h, mean 5 h). Blood samples were immediately centrifuged, and the plasma aliquots were frozen at −80°C. Cholesterol, triglycerides, high-density lipoprotein (HDL), and low-density lipoprotein (LDL) concentrations were measured from serum using enzymatic reagents (DiaSys, Holzheim, Germany) and were calibrated using secondary standards (Roche Diagnostics, Mannheim, Germany). High-sensitive C-reactive protein was measured using a particle enhanced immunoturbidimetric assay (Diasys, Holzheim, Germany). Measurements were performed on an Olympus AU680 automatic analyzer (Beckman Coulter, Brea, CA, United States). HbA1c was quantified from whole blood by high pressure liquid chromatography using D-10 (Bio-Rad, Hercules, CA, United States). NT-proBNP was determined using a chemiluminescent microparticle immunoassay (Architect, Abott, IL, United States). All tests were performed according to the manufacturer’s recommendations.

#### Cardiovascular Endurance Domain

A cardiopulmonary exercise test (CPET) until maximal exertion was performed using an electromagnetically braked cycle ergometer (Ergoselect 200; Ergoline, Bitz, Germany) and applying one of the following five ramp protocols: (i) a 3-min warm-up either unloaded, a load of 10 or 20 W for protocols 1 to 3, or a load of 50 W for protocols 4 and 5 followed by (ii) a ramp protocol with a linear workload increases of 7, 10, 15, 20, or 30 W/min for protocols 1–5, respectively, followed by (iii) a 3-min recovery phase at the same workload as the warm-up. The protocol was chosen to achieve a duration of approximately 10 min. Gas exchange and ventilatory variables were analyzed continuously (breath-by-breath) using a computer-based system (MetaMax 3B; Cortex Biophysik GmbH, Leipzig, Germany). All tests were continued until maximal exertion (i.e., volitional exertion, dyspnea, or fatigue). Before and during the test, patients were encouraged to reach their level of maximal exhaustion. Peak oxygen uptake (peak V̇O_2_) was defined as the highest 30-s average of V̇O_2_ at any point during the test. V̇O_2_ off-kinetics were assessed from the active recovery period that directly followed the incremental phase of the CPET. A complete description of the CPET is described by Wagner et al. (2020).

#### Muscle Strength and Power Domain

Isometric lower body strength was measured performing a mid-thigh pull using an analog dynamometer (TTM Muscular Meter, Tokyo, Japan). Countermovement jumps (CMJ) were performed on a force plate (Leonardo Mechanograph^®^, Novotec Medical, Pforzheim, Germany) to measure peak power and jump height. Maximal strength and rate of force development (RFD) of the handgrip were measured on the dominant side using a handheld dynamometer (Leonardo Mechanograph GF; Novotec Medical GmBH, Pforzheim, Germany).

#### Neuromuscular Coordination Domain

Balance was assessed by the path length of the center of pressure during an upright static tandem stance using the same force plate as for the CMJ. Gait parameters were assessed during habitual walking speed on a 20-m walkway using an inertial sensor system (Physilog^®^; GaitUp, Lausanne, Switzerland).

#### Physical Activity Domain

Physical Activity (PA) was measured continuously over 14 days using a wrist-worn triaxial accelerometer (GeneActive Activinsights Ltd., Kimbolton, United Kingdom). The device was attached to the participant’s non-dominant wrist and sampled data at a frequency of 50 Hz. The numbers of minutes per day performed at light (1.5–3.99 METS; metabolic equivalent of task), moderate (4.00–6.99 METS), and vigorous (≥7 METS) PA were averaged for all valid days (Esliger et al., 2011).

The exact sequence and detailed description of methods of the various measurements beyond the explanations above are described elsewhere (Wagner et al., 2019).

### Statistical Analysis

Participant characteristics were analyzed descriptively. The distribution of continuous variables was inspected graphically and characterized by means and standard deviations. Categorical variables are presented as absolute and relative frequencies. *P*-values ≤ 0.05 were considered statistically significant (two-tailed tests were performed if not otherwise specified).

#### Analyses of Health Distance

The HD is the composite measure constructed from a set of biomarkers as recently suggested (Cohen et al., 2013). It is also known as the measure of physiological dysregulation (Arbeev et al., 2019). This is the Mahalanobis distance (Mahalanobis, 1936; De Maesschalck et al., 2000) defined for vectors of biomarker measurements and it quantifies the deviations of individuals’ biomarker profiles from “optimal” (or “reference”) values in a “reference population.” This “reference population” can be represented by a subsample from the same study or some other sample can be used for this purpose. For a (column) vector of biomarkers measured in an individual *i*, *x*_*i*_, the health distance *HD*_*i*_ is defined as $H\,D_{i}=\sqrt{{\left(x_{i}-\bar{x}\right)}^{T}\,S^{-1}\,\left(x_{i}-\bar{x}\right)}$, where $\bar{x}$ is a vector of means and *S* is the variance-covariance matrix of the respective biomarkers calculated in the “reference” population (superscript *T* denotes transposition).

In this study, we constructed different variants of HDs based on the subset of biomarkers available in the COmPLETE study. As it is well known that females and males may have very different values and dynamics of many biomarkers, we constructed HDs separately for females and males as described below. The initial list of 59 biomarkers is shown in Tables 2, 3. We excluded four biomarkers from the initial list since they were included in the in- or exclusion criteria (BMI, rest systolic and diastolic BP, and NT-proBNP). In addition, the following biomarkers were excluded due to their high correlation to other biomarkers (absolute values of pairwise correlations exceeding 0.9): height, lean body mass, FEV1, LDL cholesterol, peak V̇O_2_ (mL/kg lean mass/min), peak workload, and peak V̇E. The selection of one variable within correlated groups of variables was based on previous evidence and guidelines (Lloyd-Jones et al., 2010; Wagner et al., 2018). Selected parameters are, therefore, more likely to be associated with aging, general health outcomes, or HF.

The resulting list of 48 biomarkers was included in the “All Biomarkers HD” and we also computed HDs from domain-specific sets of biomarkers indicated in Tables 2, 3 (Anthropometry; Vascular and respiratory health; Blood testing; Cardiovascular endurance; Muscle strength/power; Neuromuscular coordination; and PA). In addition, we used respective Cardiovascular endurance, Muscle strength, Neuromuscular coordination, and PA biomarkers to construct the “Fitness biomarkers HD,” and Anthropometry, Blood, and Vascular and respiratory health biomarkers to compute the “General health” HD.

As there were missing values in biomarkers (see Tables 2, 3), we performed multiple imputation of missing values of biomarkers using the R-package “mice” (Van Buuren and Groothuis-Oudshoorn, 2011). We generated 25 datasets with imputed values of biomarkers and computed HDs in each dataset using the observed and imputed values as described below (see also section “Sensitivity Analyses” regarding different imputation methods).

Prior to computations of HDs, biomarker values were transformed using the Box–Cox transformation and standardized to be on the same scale (mean = 0 and variance = 1) so that they would resemble a standard normal distribution. For biomarkers with negative values, the observations were shifted by adding a constant so that the values would be in the positive range. For computations of HDs, we selected healthy individuals younger than 40 years as the “reference population.” This cutoff resulted in a reasonably large reference population and a sizable healthy group (see also section “Sensitivity Analyses” regarding different definitions of the reference population). In each imputed dataset, we computed the means and the variance-covariance matrix in this “reference population” separately for females and males and used them in constructing HDs from observed and imputed values of biomarkers for each individual of respective sex as in the above formula. The original HDs were then transformed using the Box–Cox transformation and standardized to a zero mean and a unit variance. Note that the original HDs are positive numbers by construction (see the formula above) whereas the Box–Cox transformed ones have negative values. Thus, zero values of HDs in respective figures can be viewed as the average values of the HDs in the sample.

For each computed HD, we fitted the linear regression model with HD as the dependent variable and the disease status (0—healthy, 1—HF), sex (0—male, 1—female), age (we computed it as age—40 but refer to as “age” throughout the text for conciseness), age^2^, smoking status (0—never smoked, 1—ever smoked), medication use (0—do not use, 1—use medications indicated in Table 1), and the interaction term for the disease status and age as independent variables (see also section “Sensitivity Analyses” regarding different specifications of the regression model). The output from the analyses in each imputed dataset was pooled using the standard Rubin’s rules. The pooled estimates were used to compute the estimated values of HDs from the respective regression equation in each stratum of the dichotomous variables and for ages in the range from 40 to 91. The age trajectories of HDs for healthy and HF corresponding to the “female non-smokers not taking medications” stratum are reported in respective figures.

**TABLE 1:** Descriptive characteristics of the study population separated into Reference Population (healthy participants aged ≤39 years), Healthy (healthy participants aged ≥40 years), and Heart Failure (patients with heart failure). Data are presented as mean ± standard deviation if not stated otherwise.

Characteristics	*N*	Reference population	*N*	Healthy	*N*	Heart failure	*N*	Total sample
Participants, *n* (%)		152 (25.1)		374 (61.8)		79 (13.1)		605 (100.0)
Sex (m/f), *n* (%)	152	83 (54.6)/69 (45.4)	374	190 (50.8)/184 (49.2)	79	64 (81.0)/15 (19.0)	605	337 (55.7)/268 (44.3)
Age (years)	152	29.6 ± 5.3	374	63.9 ± 13.7	79	66.2 ± 13.3	605	55.6 ± 19.3
**NYHA class, *n* (%)**								
I	152	0 (0.0)	374	0 (0.0)	79	37 (46.8)	605	37 (6.1)
II	152	0 (0.0)	374	0 (0.0)	79	28 (35.4)	605	28 (4.6)
III	152	0 (0.0)	374	0 (0.0)	79	14 (17.7)	605	14 (2.3)
**Smoking status, *n* (%)**								
Smokers	152	0 (0.0)	374	0 (0.0)	72	7 (8.9)	598	7 (1.2)
Never smoked	152	142 (93.4)	374	280 (74.9)	72	38 (48.1)	598	460 (76.0)
Ex-smokers >10 years	152	10 (6.6)	374	94 (25.1)	72	27 (34.2)	598	131 (21.7)
**Medication use, *n* (%)**								
Medication	152	0 (0.0)	374	77 (20.6)	79	75 (94.9)	605	152 (25.1)
Antihypertensives	152	0 (0.0)	374	56 (15.0)	79	75 (94.9)	605	131 (21.7)
ACE ARB	152	0 (0.0)	374	44 (11.8)	79	65 (82.3)	605	109 (18.0)
Beta-blockers	152	0 (0.0)	374	12 (3.2)	79	58 (73.4)	605	70 (11.6)
Anticoagulants	152	0 (0.0)	374	20 (5.3)	79	69 (87.3)	605	89 (14.7)
Statins	152	0 (0.0)	374	22 (5.9)	79	62 (78.5)	605	84 (13.9)
Antidiabetics	152	0 (0.0)	374	0 (0.0)	79	14 (17.7)	605	14 (2.3)

*NYHA class, New York Heart Association functional classification; ACE, angiotensin-converting-enzyme; ARB, AT1-receptor-blocker.*

**TABLE 2:** Descriptive statistics of the male participants separated into Reference Population (healthy participants aged ≤39 years), Healthy (healthy participants aged ≥40 years), and Heart Failure (patients with heart failure). Data are presented as mean ± standard deviation if not stated otherwise.

Biomarkers	*N*	Reference population	*N*	Healthy	*N*	Heart failure	*N*	Total sample	Included in HD
**Anthropometry**									
Height (cm)	83	179.76.7	190	175.97.2	64	175.35.7	337	176.77.0	0
Body mass (kg)	83	76.79.9	190	76.69.5	64	86.814.3	337	78.611.4	1
BMI (kg/m^2^)	83	23.72.3	190	24.72.5	64	28.24.1	337	25.13.2	0
WHR	83	0.90.0	190	0.90.1	64	1.00.1	337	0.90.1	1
Body fat (%)	82	165	187	227	64	306	333	228	1
Lean body mass (kg)	82	64.78.1	187	59.87.4	64	60.57.5	333	61.17.9	0
Skeletal muscle mass (kg)	82	36.84.9	187	33.34.5	64	33.64.6	333	34.24.9	1
**Vascular and respiratory health**									
Rest systolic BP (mmHg)	83	12711	190	13113	64	12715	337	12913	0
Rest diastolic BP (mmHg)	83	748	190	818	64	7812	337	799	0
HR at rest (bpm)	83	6211	188	609	64	6313	335	6110	1
baPWV (m/s)	83	10.61.0	190	13.52.8	64	13.62.8	337	12.82.8	1
CAVI	83	6.20.9	190	8.91.6	64	9.31.6	337	8.31.9	1
Preejection period (ms)	83	96.817.7	189	104.515.7	62	122.127.6	334	105.920.6	1
Ejection time LV (ms)	83	301.716.4	190	308.721.6	64	301.934.0	337	305.723.6	1
FVC	78	5.70.8	177	4.50.9	50	3.90.8	305	4.71.1	1
FEV1	78	4.50.6	177	3.40.7	50	3.00.7	305	3.60.9	0
**Blood testing**									
NTproBNP (pg/mL)	81	58.756.1	190	121.4295.3	63	539.8535.8	334	185.1365.7	0
HbA1c (mg/dL)	82	5.00.3	189	5.30.4	63	6.10.7	334	5.40.6	1
Total cholesterol (mg/dL)	82	19035	190	22437	63	16538	335	20543	1
Triglyceride (mg/dL)	82	13280	190	12456	63	13486	335	12869	1
HDL cholesterol (mg/dL)	82	55.110.0	190	60.912.6	63	50.38.6	335	57.512.1	1
LDL cholesterol (mg/dL)	82	103.521.9	190	127.225.3	63	87.523.7	335	114.029.0	0
C-reactive protein (mg/L)	82	1.815.23	190	1.933.14	63	3.556.32	335	2.214.48	1
Creatinine (mg/dl)	82	0.890.13	190	0.920.17	63	1.130.45	335	0.950.26	1
**Cardiovascular endurance**									
Peak V̇O_2_ (L/min)	83	3.480.59	190	2.650.75	64	1.910.54	337	2.720.85	1
Peak V̇O_2_ (mL/kg/min)	83	45.97.7	190	35.09.9	64	22.16.1	337	35.211.7	1
Peak V̇O_2_ (mL/kg leanmass/min)	82	54.17.5	187	44.110.1	64	31.47.6	333	44.111.7	0
Peak O_2_ pulse (mL/beat)	83	18.72.7	183	16.73.3	64	15.33.7	330	16.93.4	1
Peak workload (W)	83	30061	190	22378	64	13947	337	22687	0
V̇O_2_ at VT1 (mL/kg/min)	83	26.16.3	190	21.06.3	63	13.64.3	336	20.97.2	1
V̇O_2_ at VT1 (L/min)	83	1.980.46	190	1.600.47	63	1.160.36	336	1.610.52	1
PETCO_2_ at rest (mmHg)	83	34.02.6	190	31.33.2	64	29.43.1	337	31.63.4	1
PETCO_2_ at VT1 (mmHg)	83	45.03.8	190	40.14.1	63	35.83.8	336	40.55.0	1
V̇E/V̇CO_2_ slope	83	35.05.5	190	37.56.8	64	42.08.5	337	37.77.2	1
V̇E/V̇CO_2_ slope below VT2	83	26.63.9	163	30.55.1	64	37.38.0	310	30.96.6	1
OUES (mL/min)	83	3431628	190	2803767	64	2111619	337	2826829	1
OUES (mL/min/kg)	83	45.28.5	190	36.910.4	64	24.36.4	337	36.511.5	1
% rel V̇O_2_ reduction 60 s post test	71	33.66.7	185	28.68.5	63	23.18.8	319	28.68.8	1
Slope linear V̇O_2_ off-kinetics (mL/min/s)	71	−22.38.1	185	−15.67.5	63	−10.05.0	319	−16.08.2	1
Peak Lac (mmol/L)	68	11.12.0	173	7.72.6	59	4.71.6	300	7.83.1	1
Peak V̇E (L/min)	83	14828	190	11734	64	8928	337	11937	0
Peak HR (bpm)	83	1908	182	16321	64	13523	329	16526	1
HRR 1 min (bpm)	78	−248	182	−249	62	−2210	322	−239	1
HRR 2 min (bpm)	79	−5914	182	−5215	62	−4819	323	−5316	1
Peak exercise systolic BP (mmHg)	73	19118	167	19725	64	16931	304	18927	1
**Muscle strength/power**									
CMJ peak power (kN)	83	3.50.6	185	2.50.6	62	2.40.8	330	2.70.8	1
CMJ height (m)	83	0.300.05	185	0.190.06	62	0.140.06	330	0.210.08	1
Hand grip strength (N)	82	491.490.8	185	414.383.0	61	398.780.1	328	430.791.4	1
Hand grip RFD (N/150 ms)	82	327.478.0	185	273.782.1	61	203.862.0	328	274.187.4	1
Isometric leg strength (kg)	83	16131	189	13035	62	11831	334	13637	1
**Neuromuscular coordination**									
COP path length (cm)	83	25.06.9	182	44.525.9	60	48.320.8	325	40.223.4	1
Gait speed (m/s)	77	1.40.2	178	1.40.2	64	1.30.2	319	1.40.2	1
Gait cadence (steps/minute)	77	1107	178	1126	64	1108	319	1117	1
Stride length (m)	77	1.530.11	178	1.480.14	64	1.410.15	319	1.480.14	1
Gait double support (%)	77	22.12.7	178	22.02.9	64	22.43.7	319	22.13.0	1
Gait asymmetry (%)	77	2.42.3	178	2.93.4	64	2.72.8	319	2.73.1	1
**Physical activity**									
Light physical activity (min/day)	80	9328	183	9328	58	8836	321	9230	1
Moderate physical activity (min/day)	80	17952	183	15260	58	10758	321	15162	1
Vigorous physical activity (min/day)	80	1011	181	812	58	24	319	811	1

*HD, health distance; BMI, body mass index; WHR, waste-to-hip ratio; BP, blood pressure; HR, heart rate; baPWV, brachial-ankle pulse wave velocity; CAVI, cardio–ankle vascular index; LV, left ventricular; FVC, forced vital capacity, FEV1, forced expiratory volume in 1 second; LDL, low-density lipoprotein; HDL, high density lipoprotein; VO_2max_, maximal oxygen uptake; VT, ventilatory threshold; P_ET_CO_2_, partial pressure of end-tidal CO_2_; VCO_2_, carbon dioxide output; VE, volume of expiration; OUES, oxygen uptake efficiency slope; Lac, lactate; HRR, heart rate recovery; CMJ, counter movement jump; RFD, rate of force development, COP, center of pressure.*

**TABLE 3:** Descriptive characteristics of the female participants separated into Reference Population (healthy participants aged ≤39 years), Healthy (healthy participants aged ≥40 years), and Heart Failure (patients with heart failure). Data are presented as mean ± standard deviation if not stated otherwise.

Biomarkers	*N*	Reference population	*N*	Healthy	*N*	Heart failure	*N*	Total sample	Included in HD
**Anthropometry**									
Height (cm)	69	168.25.9	184	164.77.3	15	161.76.7	268	165.47.1	0
Body mass (kg)	69	62.49.0	184	62.78.7	15	72.417.1	268	63.29.6	1
BMI (kg/m^2^)	69	22.02.9	184	23.12.6	15	27.66.1	268	23.13.2	0
WHR	69	0.80.1	184	0.80.1	15	0.90.1	268	0.80.1	1
Body fat (%)	69	237	182	297	15	396	266	288	1
Lean body mass (kg)	69	47.34.6	182	44.76.4	15	43.37.6	266	45.36.2	0
Skeletal muscle mass (kg)	69	26.12.8	182	24.33.9	15	23.24.4	266	24.73.7	1
**Vascular and respiratory health**									
Rest systolic BP (mmHg)	69	11410	184	12814	15	13016	268	12415	0
Rest diastolic BP (mmHg)	69	718	184	789	15	7710	268	769	0
HR at rest (bpm)	69	619	184	6310	15	6314	268	6210	1
baPWV (m/s)	69	9.80.8	184	13.02.6	15	13.22.7	268	12.22.7	1
CAVI	69	6.20.8	184	8.61.4	15	8.71.5	268	8.01.6	1
Preejection period (ms)	68	101.317.1	184	107.218.3	15	119.426.7	267	106.418.9	1
Ejection time LV (ms)	69	314.713.2	184	317.721.8	15	314.737.9	268	316.821.1	1
FVC	61	4.10.6	169	3.20.7	12	2.30.5	242	3.40.8	1
FEV1	61	3.40.5	169	2.50.6	12	1.90.4	242	2.70.7	0
**Blood testing**									
NTproBNP (pg/mL)	64	105.395.3	181	155.4163.7	14	887.71084.5	259	182.6330.4	0
HbA1c (mg/dL)	65	5.00.3	182	5.30.4	14	5.80.3	261	5.30.4	1
Total cholesterol (mg/dL)	65	19234	181	24040	14	19939	260	22644	1
Triglyceride (mg/dL)	65	8841	181	11562	14	162112	260	11163	1
HDL cholesterol (mg/dL)	65	70.815.6	181	73.513.8	14	62.813.5	260	72.314.4	1
LDL cholesterol (mg/dL)	65	101.421.3	181	134.027.8	14	103.825.3	260	124.230.0	0
C-reactive protein (mg/L)	65	1.322.21	181	1.923.49	14	4.103.63	260	1.893.26	1
Creatinine (mg/dl)	65	0.740.10	181	0.760.13	14	0.850.19	260	0.760.13	1
**Cardiovascular endurance**									
Peak V̇O_2_ (L/min)	69	2.430.38	184	1.780.52	15	1.190.30	268	1.910.58	1
Peak V̇O_2_ (mL/kg/min)	69	39.46.6	184	28.47.3	15	16.95.0	268	30.69.1	1
Peak V̇O_2_ (mL/kg leanmass/min)	69	51.57.1	182	39.58.4	15	27.66.6	266	42.010.1	0
Peak O_2_ pulse (mL/beat)	64	13.82.7	179	11.43.1	15	9.52.9	258	11.93.2	1
Peak workload (W)	69	20535	184	14055	15	7622	268	15360	0
V̇O_2_ at VT1 (mL/kg/min)	69	23.35.3	184	17.94.5	15	10.92.1	268	18.95.5	1
V̇O_2_ at VT1 (L/min)	69	1.440.29	184	1.120.32	15	0.780.23	268	1.190.36	1
PETCO_2_ at rest (mmHg)	69	30.92.0	184	30.72.7	15	28.82.1	268	30.62.5	1
PETCO_2_ at VT1 (mmHg)	69	42.13.5	184	39.44.0	15	34.72.7	268	39.84.2	1
V̇E/V̇CO_2_ slope	69	33.95.7	184	37.06.3	15	41.37.5	268	36.46.5	1
V̇E/V̇CO_2_ slope below VT2	69	27.04.1	152	30.55.1	15	40.17.7	236	30.15.9	1
OUES (mL/min)	69	2549492	184	1934556	15	1437485	268	2064617	1
OUES (mL/min/kg)	69	41.18.9	184	30.98.2	15	19.86.5	268	32.99.9	1
% rel V̇O_2_ reduction 60 s post test	68	34.36.4	179	28.17.9	15	15.39.7	262	28.98.8	1
Slope linear V̇O_2_ off-kinetics (mL/min/s)	66	−17.25.0	180	−11.05.1	15	−4.92.7	261	−12.25.9	1
Peak Lac (mmol/L)	59	9.32.1	155	6.32.2	13	4.11.5	227	6.92.6	1
Peak V̇E (L/min)	69	10319	184	7823	15	5112	268	8325	0
Peak HR (bpm)	63	1869	179	16119	15	13720	257	16621	1
HRR 1 min (bpm)	64	−2510	174	−2510	15	−208	253	−2510	1
HRR 2 min (bpm)	64	−5917	171	−5216	15	−4620	250	−5317	1
Peak exercise systolic BP (mmHg)	58	17020	153	18421	15	17433	226	18023	1
**Muscle strength/power**									
CMJ peak power (kN)	68	2.20.4	177	1.60.4	15	1.40.4	260	1.80.5	1
CMJ height (m)	68	0.210.04	177	0.130.05	15	0.070.03	260	0.150.06	1
Hand grip strength (N)	68	317.854.6	179	271.460.9	15	238.238.8	262	281.562.5	1
Hand grip RFD (N/150 ms)	68	224.846.0	179	178.356.5	15	129.037.5	262	187.558.4	1
Isometric leg strength (kg)	69	10525	184	7826	14	5523	267	8429	1
**Neuromuscular coordination**									
COP path length (cm)	67	22.69.0	175	39.420.8	14	45.418.0	256	35.419.8	1
Gait speed (m/s)	68	1.40.1	173	1.40.2	14	1.30.1	255	1.40.2	1
Gait cadence (steps/minute)	68	1167	173	1188	14	1208	255	1188	1
Stride length (m)	68	1.480.10	173	1.430.14	14	1.290.10	255	1.430.13	1
Gait double support (%)	68	21.72.7	173	21.03.0	14	22.02.8	255	21.22.9	1
Gait asymmetry (%)	68	1.91.8	173	2.42.1	14	2.72.5	255	2.32.1	1
**Physical activity**									
Light physical activity (min/day)	68	9523	175	11432	15	9838	258	10831	1
Moderate physical activity (min/day)	68	17747	175	16163	15	11273	258	16361	1
Vigorous physical activity (min/day)	67	88	174	45	15	11	256	57	1

*HD, health distance; BMI, body mass index; WHR, waste-to-hip ratio; BP, blood pressure; HR, heart rate; baPWV, brachial-ankle pulse wave velocity; CAVI, cardio–ankle vascular index; LV, left ventricular; FVC, forced vital capacity, FEV1, forced expiratory volume in 1 second; LDL, low-density lipoprotein; HDL, high density lipoprotein; VO_2max_, maximal oxygen uptake; VT, ventilatory threshold; P_ET_CO_2_ = partial pressure of end-tidal CO_2_; VCO_2_, carbon dioxide output; VE, volume of expiration; OUES, oxygen uptake efficiency slope; Lac, lactate; HRR, heart rate recovery; CMJ, counter movement jump; RFD, rate of force development, COP, center of pressure.*

We also fitted the logistic regression model for the disease status as the outcome and sex, age, and age^2^ as covariates in the base (reference) model and the same covariates plus combinations of one or two HDs (HD1 only, HD2 only, HD1 and HD2; for specific types of HD1 and HD2, see section “Results”) to compare the performance of different models in predictions of HF cases. Neuromuscular coordination was not included in the area under the receiver operating characteristic (ROC) curve (AUC) analysis due to the non-significant HD difference between Healthy and HF. We evaluated the AUC’s and differences between those, along with values of sensitivities and specificities, in each imputed dataset. Leave-one-out cross-validation was used for model evaluation in each respective calculation. We reported median values and interquartile ranges of AUCs across all imputed datasets and estimated differences in AUC pooled using the standard Rubin’s rules. The ROC curves in each imputed dataset and the ROC curve drawn at median values of 1—specificity and sensitivity across all imputed datasets are presented in the main text and in the Supplementary Material.

#### Sensitivity Analyses

We performed sensitivity analyses to check sensitivity of results to various aspects of computational workflows which could hypothetically affect the estimates and conclusions. First, we used different specifications of imputation models for biomarkers in the multiple imputation procedure: (a) age, sex, disease status; (b) age, sex; (c) age, sex, disease status, biomarkers; (d) age, sex, biomarkers; (e) age, sex, disease status, biomarkers, other covariates (such as smoking, medications); (f) age, sex, biomarkers, other covariates. All results were similar in all imputation methods. Therefore, we report only the results for option a) in the paper. Second, we checked another cutoff to define the reference population (younger than 50 years). We replicated all results using the same models but with age_50 (computed as age—50) and all conclusions were identical. Hence, only the baseline scenario with the cutoff age 40 is reported. Third, we checked other sets of covariates in the linear regression model (excluding smoking, medication, age^2^, and the interaction term for the disease status and age). All estimates for the disease status variable were qualitatively similar in all models. We also tested the model with the interaction term for disease status and age^2^ and the results for the disease status were similar to the model without this term. As the regression coefficient for the interaction term for the disease status and age^2^ was not significant, we reported the model without this term.

Descriptive analyses, construction of HDs, linear regression analyses, and tabulation of results were performed in R version 3.6.1 or later and in MATLAB R2019b. Logistic regression analyses were done in SAS 9.4 (SAS/STAT 14.3). MATLAB R2019b was used for visualization.

## Results

### Participant Characteristics

A total of 526 healthy participants and 79 cardiac patients with HF (NYHA functional classes I–III) were included. All HF patients were in a stable condition; their etiology was cardiomyopathy (*n* = 8), coronary artery disease (*n* = 60), pulmonary hypertension (*n* = 1), valvular regurgitation (*n* = 8) or valvular stenosis (*n* = 2). Subjects’ characteristics and biomarkers are presented in Tables 1–3 stratified by subgroups including young healthy individuals ≤39 years of age (Reference Population), healthy individuals ≥40 years of age (Healthy), and patients with HF. Table 1 indicates that participants from the HF group are approximately 2.3 years older on average compared to participants in the Healthy group (*p* = 0.16). The proportion of females in the both the Reference Population and the Healthy group is nearly 50%, whereas the proportion of females in the HF group is 19%. Medication use differs between the Reference Population, the Healthy group, and the HF group and is most prevalent in the HF group. Differences described above are significant (*p* < 0.0001).

Tables 2, 3 present descriptive statistics for the biomarkers selected for computations of HD. Most of the biomarkers included in the HD calculation were highly correlated with age (*p* < 0.0001) (Supplementary Table S1). The mean values of biomarkers differed between the reference population (≤39 years of age) and the Healthy group (≥40 years of age) in most biomarkers (*p* < 0.0001) (Supplementary Table S2). We note, however, that these results are purely descriptive and do not explore how multiple factors (except age) may contribute to such differences.

### Health Distances in Health and Heart Failure

**FIGURE 1:**
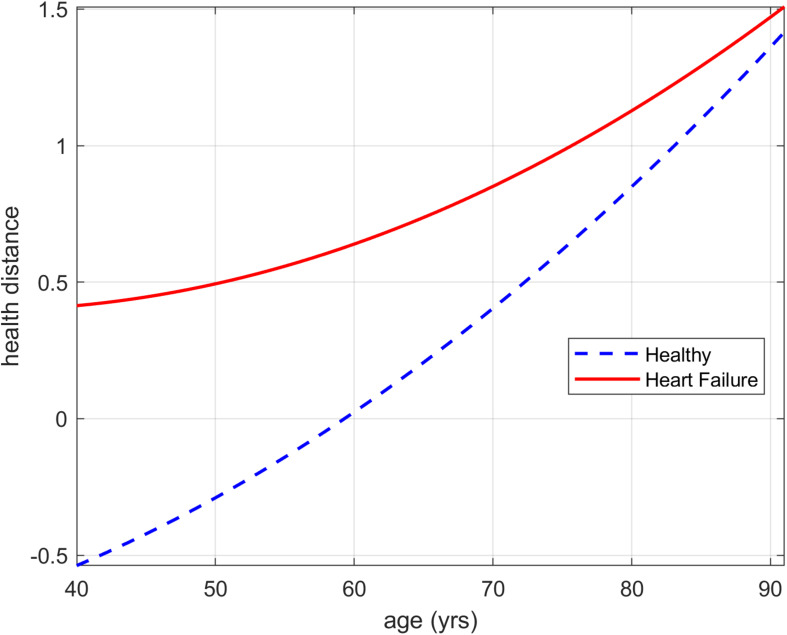
Health distance trajectories for Fitness biomarkers for the Healthy and Heart Failure group presented from 40 to 91 years of age. The curves correspond to non-smoking females not taking medications.

Group differences between Healthy individuals ≥40 years of age and patients with HF are reported in Table 4. Nine out of 10 HD showed evidence for a difference between the groups (*p* ≤ 0.002). HD trajectories for *Fitness* for the Healthy and HF group are presented from 40 to 91 years in Figure 1. Trajectories of additional HD can be found in Supplementary Figures S1–S9. The HD trajectories of the healthy group continuously increase starting with negative values to values of >1.3 (see section “Analyses of Health Distance” regarding interpretation of zero HD). In contrast, the HF group HD trajectories already begin at a positive HD of approximately 0.4 and increase to a similar region as the healthy counterparts’ HD reaching values of >1.4 at 91 years. The largest HD difference between those groups is observed at the youngest age (40 years) after that the HD trajectories of *Fitness* of the Healthy and HF group continuously approach each other with increasing age. The approaching pattern of HD is observed in most of the calculated HDs presented in Table 4 (see Supplementary Figures S1–S9).

**TABLE 4:** Estimates of group differences in Health Distance for Healthy (healthy participants aged ≥40 years) and Heart Failure (patients with heart failure). Estimates and standard errors are for age 40 years.

Health distance	Estimate	SE	*p*-value	*Adjusted p*-value
All biomarkers	1.05	0.15	<0.0001	<0.0001
Fitness biomarkers	0.95	0.17	<0.0001	<0.0001
Cardiovascular endurance biomarkers	1.44	0.18	<0.0001	<0.0001
Muscles strength biomarkers	1.36	0.21	<0.0001	<0.0001
Neuromuscular coordination biomarkers	0.26	0.26	0.3047	1.0000
Physical activity biomarkers	1.07	0.29	0.0002	0.0020
General health biomarkers	1.48	0.17	<0.0001	<0.0001
Anthropotmetry biomarkers	1.49	0.27	<0.0001	<0.0001
Blood biomarkers	1.66	0.27	<0.0001	<0.0001
Vascular and respiratory health biomarkers	1.15	0.16	<0.0001	<0.0001

*Column “adjusted *p*-value” reports *p*-values adjusted for multiple comparisons using the Bonferroni correction.*

### Predicting HF Cases Using Health Distances

**FIGURE 2:**
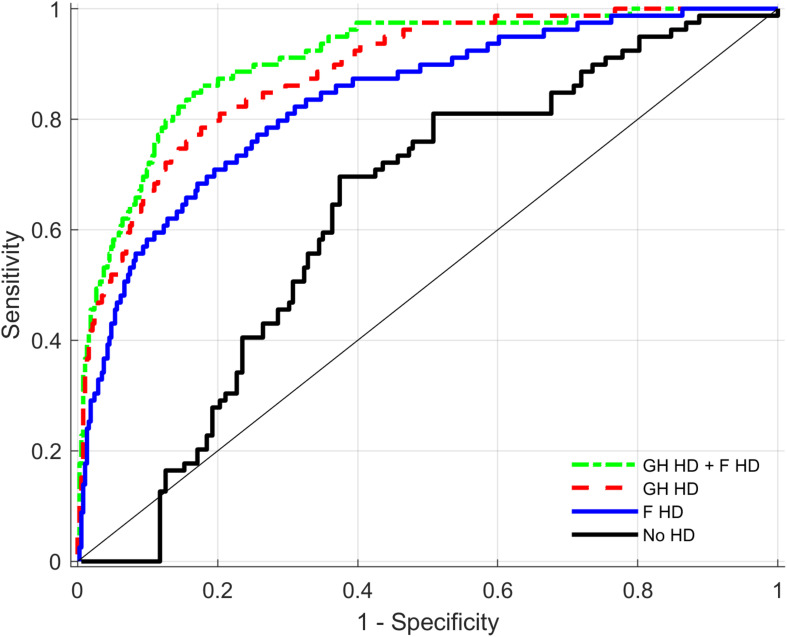
Receiver operating characteristics (ROC) curves for health distances (HD) of General health (GH), Fitness (F), and the combination of both HDs as predictors of Heart Failure. ROC curves present combined output from all imputed datasets (see section “Materials and Methods”).

**FIGURE 3:**
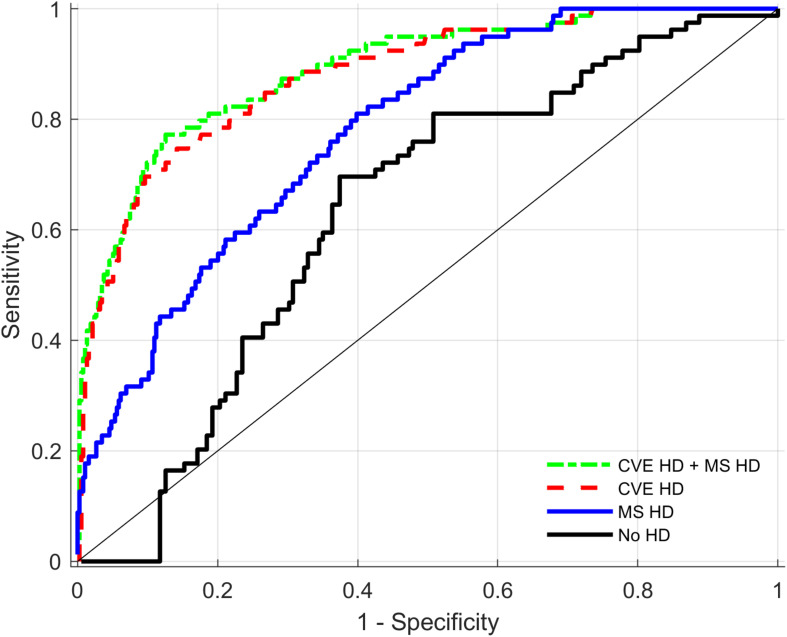
Receiver operating characteristics (ROC) curves for Health distances (HD) of Cardiovascular endurance (CVE), Muscle strength (MS), and the combination of both HDs as predictors of Heart Failure. ROC curves present combined output from all imputed datasets (see section “Materials and Methods”).

**FIGURE 4:**
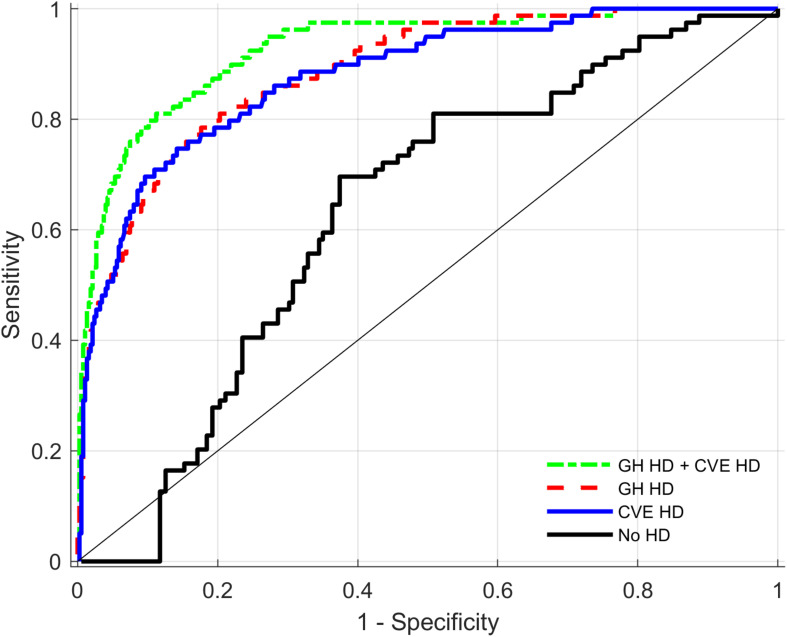
Receiver operating characteristics (ROC) curves for Health distances (HD) of General health (GH), Cardiovascular endurance (CVE) and the combination of both HDs as predictors of Heart Failure. ROC curves present combined output from all imputed datasets (see section “Materials and Methods”).

Discriminative performance analyses presented in Table 5 compare the performance of different models in predictions of HF cases (cardiac disease) for the total sample. The table shows the median estimates of AUCs for three different models including different HDs and always including the base model (age, age^2^, and sex). Further differences between AUC within these models are presented. Figure 2 indicates that compared to the base model with sex and age, both the *General health* and the *Fitness* biomarkers increase the AUC estimate significantly from 0.63 to 0.89 and 0.84, respectively. In addition, there is a significant additional benefit when combining these two HDs compared to one HD alone. Compared to the initial model with sex, age and age^2^, both *Cardiovascular endurance* and *Muscle strength* biomarkers increase the AUC estimates substantially from 0.63 to 0.88 and 0.78, respectively (see also Figure 3). There is, however, little evidence that adding HD *Muscle strength* to HD *Cardiovascular endurance* adds value. Table 5 (see also Figure 4) shows that *Cardiovascular endurance* alone reaches an AUC of 0.88 compared to *General health* HD with 0.89. Further, there is evidence that the combination of both HD *Cardiovascular endurance* and HD *General health* shows superior predictive power compared to one of the HDs alone.

**TABLE 5:** Areas under receiver operating characteristics curves (AUC) in models with different health distances (HD) as predictors of Heart Failure. AUC presented as median (IQR) across all imputed datasets.

	AUC	AUC difference			
		
Model	Median (IQR)	Contrast	Mean (SE)	95% CI	*p*-value
no HD	0.632				
General health HD (GH HD)	0.886 (0.010)	GH HD – no HD	0.252 (0.033)	[0.189, 0.316]	<0.0001
Fitness HD (F HD)	0.838 (0.013)	F HD – no HD	0.202 (0.032)	[0.139, 0.266]	<0.0001
General health HD and Fitness HD	0.908 (0.006)	GH HD and F HD – no HD	0.275 (0.032)	[0.213, 0.338]	<0.0001
		GH HD and F HD – GH HD	0.023 (0.010)	[0.004, 0.042]	0.0200
		GH HD and F HD – F HD	0.073 (0.021)	[0.032, 0.114]	0.0005
		GH HD – F HD	0.050 (0.028)	[−0.004, 0.104]	0.0718
no HD	0.632				
Cardiovascular endurance HD (CVE HD)	0.877 (0.006)	CVE HD – no HD	0.246 (0.033)	[0.182, 0.311]	<0.0001
Muscle strength HD (MS HD)	0.780 (0.005)	MS HD – no HD	0.147 (0.031)	[0.086, 0.207]	<0.0001
Cardiovascular endurance HD and Muscle strength HD	0.889 (0.005)	CVE HD and MS HD – no HD	0.257 (0.033)	[0.192, 0.323]	<0.0001
		CVE HD and MS HD – CVE HD	0.011 (0.008)	[−0.005, 0.027]	0.1694
		CVE HD and MS HD – MS HD	0.111 (0.021)	[0.069, 0.153]	<0.0001
		CVE HD – MS HD	0.100 (0.026)	[0.049, 0.151]	0.0001
no HD	0.632				
General health HD (GH HD)	0.886 (0.010)	GH HD – no HD	0.252 (0.033)	[0.189, 0.316]	<0.0001
Cardiovascular endurance HD (CVE HD)	0.877 (0.006)	CVE HD – no HD	0.246 (0.033)	[0.182, 0.311]	<0.0001
General health HD and Cardiovascular endurance HD	0.927 (0.003)	GH HD and CVE HD – no HD	0.294 (0.032)	[0.232, 0.356]	<0.0001
		GH HD and CVE HD – GH HD	0.042 (0.012)	[0.017, 0.066]	0.0009.
		GH HD and CVE HD – CVE HD	0.048 (0.015)	[0.018, 0.077]	0.0015
		GH HD – CVE HD	0.006 (0.024)	[−0.042, 0.054]	0.8041

*HD, health distance; GH, general health; F, fitness; CVE, cardiovascular endurance; MS, muscle strength; IQR, interquartile range; SE, standard error; CI, confidence interval.*

## Discussion

Our study is the first to comprehensively measure all physical fitness components in both a healthy sample over the life span from 20 to 91 years and in patients with HF. Further, the study applied a novel approach from the field of epidemiology and research on aging to physiological biomarkers of physical function. Our results showed that HD composed of physical fitness or standard clinical health biomarkers differ between healthy individuals and patients with HF and that these differences reduce with increasing age. Further, HD of physical fitness can significantly increase the predictive power to detect HF cases in our sample beyond sex and age but also beyond classic clinical biomarkers.

### Differences Between Healthy Participants and Patients With HF

Age- and sex-adjusted differences between healthy individuals and patients with HF can be observed in various combinations of biomarkers summarized to composite outcomes (HD). Both HD *Fitness* and HD *General health* show evidence for a difference between groups. Out of the fitness domains, cardiovascular endurance and muscle strength but not neuromuscular performance differed between groups. The observed differences in the composite outcome *Cardiovascular endurance* and *Muscle strength* do support previous findings showing that single markers of the strength domain such as isometric leg strength or handgrip strength (Harrington et al., 1997; Izawa et al., 2007; Fulster et al., 2013) and markers of cardiovascular endurance such as peak V̇O_2_, oxygen uptake efficiency slope (OUES), or V̇E/V̇CO_2_ are reduced in patients with HF. Combining and summing several relevant biomarkers showing differences between these two groups already for themselves to one composite outcome unsurprisingly led to highly significant group differences in the present study because presumable true signals are summed. In contrast, our results did not show evidence for the composite outcome *Neuromuscular coordination* between healthy individuals and patients with HF. In the field of cardiology, measures of frailty have, however, gained attention in addition to strength and endurance performance tests (Afilalo et al., 2009). HF affects predominantly older individuals (Virani et al., 2020), and patients with HF have a higher prevalence of frailty (Newman et al., 2001; Cacciatore et al., 2005; Afilalo et al., 2009). The assessment of gait speed has been demonstrated to be a reliable single marker of frailty in older patients with HF and gait speed is independently associated with death, hospitalization for HF, all-cause hospitalization and improves risk stratification (Pulignano et al., 2016). The HD *Neuromuscular coordination* composed of gait parameters and a balance measure may not provide relevant information in our sample because the mean age of the HF group was relatively young (66 years) and measures of neuromuscular coordination are deteriorated more commonly at older age (Newman et al., 2016).

Although closely related, PA deserves a distinct glance to the other fitness outcomes because it is a behavioral measure. In line with previous evidence examining PA patterns, PA behavior seems to differ in patients with HF compared to healthy individuals (Conraads et al., 2012) and get worse with increasing clinical severity of HF (Jehn et al., 2009). The known inverse association between fitness measures and the development of HF, and the potential to increase those fitness outcomes in patients with HF by PA, provides clinicians with a powerful tool. As observed in our study, HD *Fitness* and HD *PA* are both affected by HF and often present a vicious cycle between the behavioral component and the functional outcomes.

From the *General health* HDs, particularly HD *Blood markers* and HD *Anthropometry* observed large differences between the groups. Even though only unspecific blood markers were included (because NT-proBNP was excluded due to the utilization as HF group inclusion criteria) substantial group differences were observed and were largely age independent over the lifespan from 40–91 years.

#### Trajectories

Both groups (Healthy and HF) showed a curvilinear increase in HDs, with the largest difference observed at the youngest age of approximately 40 years (Figure 1). Both Healthy and HF seem to converge toward an unknown upper limit which might indicate disability or mortality. The decline of physiological functions with advancing age including physical fitness seems to be an unstoppable process and affects these functions whether a diagnosed chronic disease is present or absent. The trend toward a highly similar HD at the highest ages might be explained by the burden to take part in such a study and by the decision of an individual or of the referring physician to enroll the patient. The upper limit of the described HDs for healthy elderly and HF might, therefore, describe the minimum level of physiological function and fitness required to be able to keep an appointment of several hours. In addition, the development of HF at an older age is probably characterized by better physical function and a better risk factor profile earlier in life, which might reduce HD between healthy individuals and patients with HF at old ages. According to our results and models, the older the individuals are, the more difficult it is to differentiate between early stages of HF and the biological effect of aging. Therefore, it can be argued that targeting physiological functions (fitness and general health functions) with increasing age is essential whether a manifest chronic disease (in our case HF) is present or absent. HD and, therefore, physiological dysregulation increases sharply with advancing age and, thus, likely decreases robustness and resilience of an individual.

### Predicting HF Cases

The fact that HD *Fitness* could detect HF patients with an estimate of AUC = 0.84 is a notable result and supports recent findings describing the importance of physical fitness assessment in clinical practice in general but specifically in patients with HF (Ross et al., 2016). Noteworthy is the finding that HD *Cardiovascular endurance* showed a higher AUC compared to HD *Fitness*, which includes the same biomarkers as *Cardiovascular endurance* but in addition the biomarkers of the fitness domains *Muscle strength* and *Neuromuscular function*. This finding indicates that including a larger number of biomarkers does not automatically improve the effect size of a composite measure consisting of physiological biomarkers. Including less but “relevant” biomarkers for the given task was superior in our dataset. Summing “relevant” biomarkers improves the effect size whereas the inclusion of less “relevant” biomarkers worsens the effects size due to adding “noise” and diluting the signal. Similar observations were made in the field of genetics where application of the concept of “polygenic risk scores,” which combine effects of different genetic markers in one combined score, often result in similar findings (Euesden et al., 2015).

The reason that HD *Muscle strength* does not add additional value to HD *Cardiovascular endurance* could be explained by noting that biomarkers included in HD *Cardiovascular endurance* such as peak V̇O_2_ (L/min) or peak lactate are likely associated with biomarkers of HD *Muscle strength* in our heterogeneous sample. Further, biomarkers included in HD *Cardiovascular endurance* might not only represent central but also peripheral limitations and muscle strength and power to some extent.

The observation that the combination of both HD *Cardiovascular endurance* and HD *General health* has superior discriminative performance than HD *General health* alone further strengthens the importance of cardiopulmonary exercise testing.

The described calculation approach of HD provides an interesting tool for future investigation and might have potential to discriminate healthy aging from the early beginning of chronic disease. It could indicate when an overall accelerated decline beyond that typically observed in healthy aging begins and, therefore, mark the optimal starting point for specific exercise interventions to prevent age-related chronic diseases. Which biomarkers and which combinations of biomarkers should be included in an optimal HD measure requires further research.

Further, HD based on multiple biomarkers represents conceptually different components of the vulnerability to age-related disease compared to values of individual biomarkers, as argued by Arbeev et al. (2019). HDs based on deviations of multiple biomarkers from their baseline states characterize the level of systemic dysregulation in physiological functions, which does not specifically require an individual biomarker to be highly abnormal or present a value typically seen in an individual with a chronic disease. For the composite measure HD, each deviation from the reference population may in principle lie within a clinically normal range; hence, the quantity and variety of biomarkers can contribute more to this composite estimate than the manifestation of any individual marker in regard of HF pathology. HD seems, particularly useful when the overall deviation of physiological functions, such as overall physical fitness, is of interest, independently of a specific disease. When a syndrome such as HF is multifaceted and impacts physical fitness over a variety of pathways, whereby both, central limitations (of the heart itself) and peripheral limitations within the skeletal musculature contribute to the overall reduction in physical functions (Houstis et al., 2018), this approach can also be promising. HDs could be less specific than a single measure such as NT-proBNP, used for diagnosis of HF, but provide an overall measure of systemic dysregulation and reduction in physical fitness and thus an indication for a therapeutic approach. We note also that the approach for constructing a composite measure based on the Mahalanobis distance which we applied in this paper is not the only technique that can be used for constructing such cumulative quantities from sets of biomarkers. For example, the methods based on the principal component analysis (Nakamura and Miyao, 2008; Kimura et al., 2012) and modifications of frailty index (Mitnitski et al., 2001) applied to biomarkers (see, e.g., Howlett et al., 2014) along with various conceptualizations of allostatic load (Seeman et al., 1997) were suggested in the literature. The choice of the approach to be used largely depends on what research questions it can help address and how the constructed measure can be interpreted in the context of the research area. Discussion on advantages and limitations of such approaches is beyond the scope of this paper.

Currently, there is a lack of physical fitness measurements in clinical practice as clinical vital signs and if assessed health care professionals pay attention to a single biomarker such as peak V̇O_2_. For this biomarker widely known cut-offs exist that correspond to clinical or preclinical manifestation of a particular disease (e.g., a peak V̇O_2_ < 20 mL/kg/min is an indication of a mild to moderate impairment of HF according to the Weber classification; Arena et al., 2014). HD, however, presents a more sophisticated approach using a cluster of abnormal values of different fitness biomarkers that occur together. The use of HD measures represents an additional step forward because it allows to utilize not only clinically “abnormal” values of physical fitness markers, but also those deviations from the baseline physiological state that, individually would not be considered as a clinically relevant reduction in physical fitness, but together may significantly contribute to the transition from healthy to unhealthy state.

Overall, this study demonstrates that a novel statistical tool, previously applied successfully in large-scale epidemiological studies using simpler biomarkers, can also be applied to physiological markers of physical function. This approach could further strengthen a comprehensive physical fitness assessment. It may help to find intervention and treatment options to decrease the accelerated decline of physiological function and, in particular, physical fitness accompanied with chronic disease and with the process of aging and thereby increase health span.

### Limitations

Our study has limitations. First, this research was cross-sectional and, therefore, no hard endpoints such as mortality or hospitalization were available. Second, the HF patient sample was rather small for investigating trajectories of HD over the age span, and the studied patients presented mostly light to moderate HF, with only a few patients with NYHA class III. Further analyses in larger population-based samples of healthy and HF individuals are needed to confirm the associations and findings observed in this study on a broader scale. For example, applications in larger studies will provide opportunity for analyses stratified by sex that can produce a more accurate picture about sex-specific differences in HDs and their relationship with respective outcomes. Applications to longitudinal studies will allow exploring associations of such HDs with mortality and morbidity risks and other time-to-event outcomes collected in such studies.

## Conclusion

Health distance composed of physical fitness biomarkers differs between healthy individuals and patients with HF and those differences between groups diminish with increasing age. In both healthy individuals and patients with HF, HD tends toward a common unknown upper limit indicating frailty, disability, or mortality. HDs can successfully predict HF cases, and HD *Cardiovascular endurance* can significantly increase the predictive power beyond classic clinical biomarkers. This study shows that a novel statistical tool from the field of epidemiology can be successfully applied to physiological biomarkers of physical function. The application of HD could strengthen a comprehensive physical fitness assessment and may present an optimal target for interventions to slow the decline of physical fitness with aging and, therefore, increase health span, and delay the onset of chronic disease.

## Data Availability Statement

The raw data supporting the conclusions of this article will be made available by the authors, without undue reservation.

## Ethics Statement

The studies involving human participants were reviewed and approved by the Ethics Committee of Northwestern and Central Switzerland (EKNZ 2017-01451). The patients/participants provided their written informed consent to participate in this study.

## Author Contributions

JW, RK, HH, TH, AS-T, and KA contributed to conceptualization. JW, RK, EL, TH, AS-T, and KA contributed to methodology. KA contributed to statistical analysis. JW, RK, KK, CK, JC, HS, and WM contributed to investigation. AS-T contributed to resources. JW and RK contributed to data curation. JW and KA contributed to writing—original draft. RK, KK, CK, JC, EL, HS, WM, HH, TH, DS, AS-T contributed to writing—review and editing. JW contributed to project administration. AS-T contributed to funding acquisition. All authors have read and approved the final manuscript.

## Conflict of Interest

WM was employed by the company SYNLAB Holding Deutschland GmbH. SYNLAB holdings Deutschland GmbH had no role in the funding, conceptualization, conduct, or interpretation of the study. The remaining authors declare that the research was conducted in the absence of any commercial or financial relationships that could be construed as a potential conflict of interest.
