# Supplementary material for: Composite Measures of Physical Fitness to Discriminate Between Healthy Aging and Heart Failure: The COmPLETE Study
Source: Front Physiol. 2020 Dec 15;11:596240. doi: 10.3389/fphys.2020.596240 (PMC7770139; doi:10.3389/fphys.2020.596240)
Supplement: Supplementary file 1 [file Table_1.DOCX]

Supplementary Material

# Supplementary Figures and Tables

## Supplementary Figures


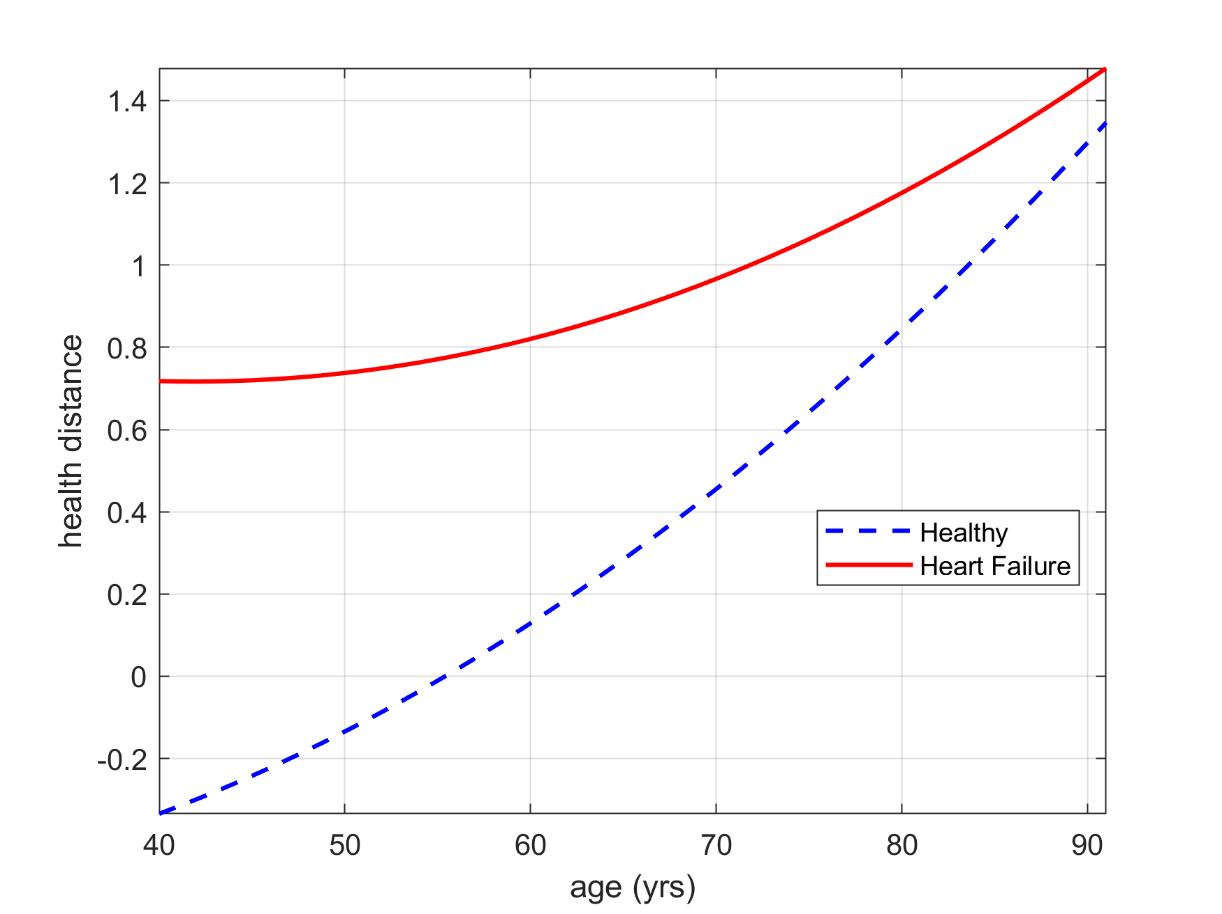


**Supplementary Figure S1.** Health distance trajectories for all biomarkers for the Healthy and Heart Failure group presented from 40 to 91 years. The curves correspond to non-smoking females not taking medications.


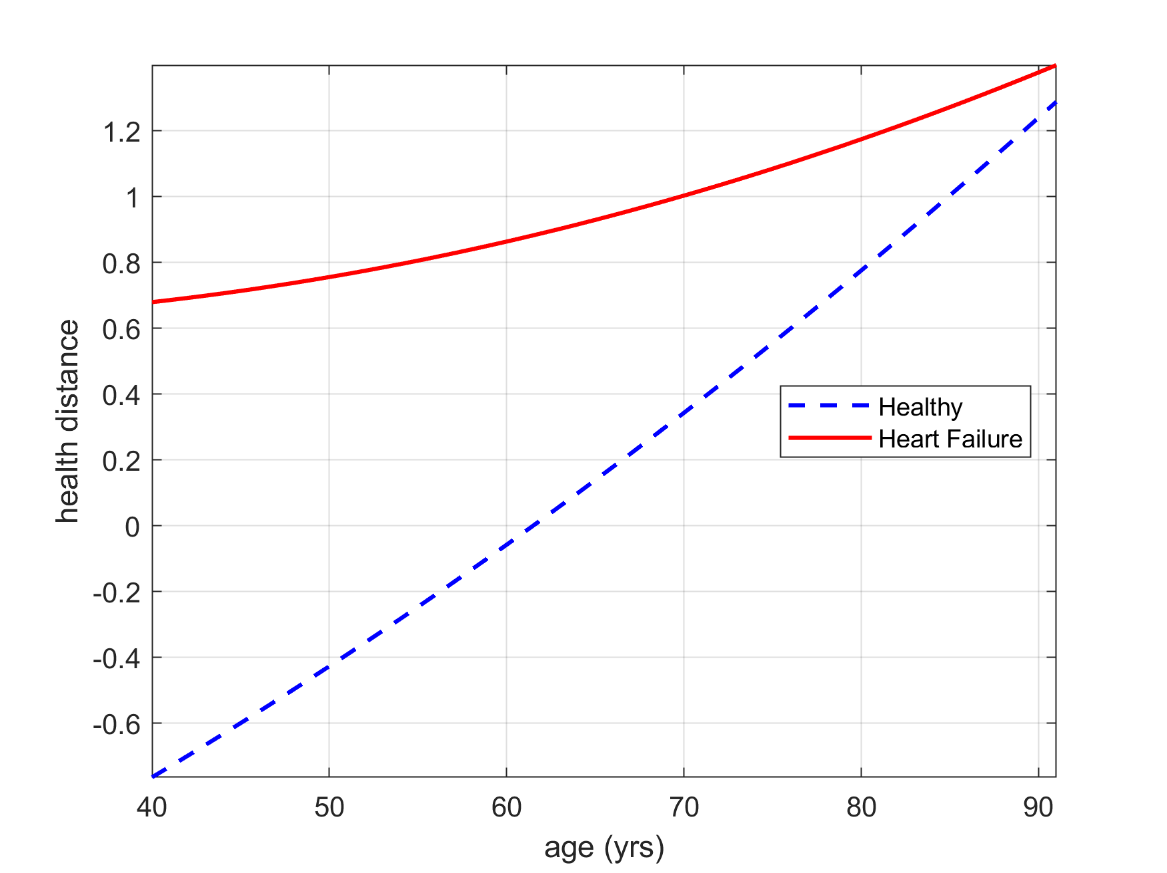


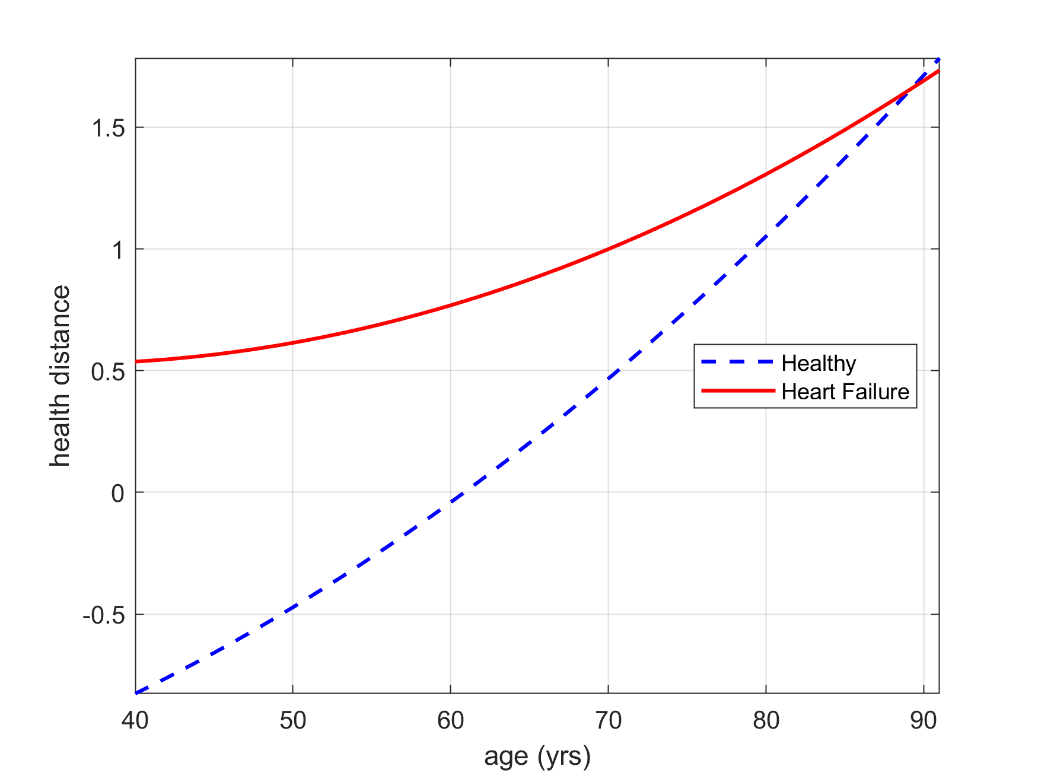
**Supplementary Figure S2.** Health distance trajectories for Cardiovascular endurance for the Healthy and Heart Failure group presented from 40 to 91 years. The curves correspond to non-smoking females not taking medications.

**Supplementary Figure S3.** Health distance trajectories for Muscle strength for the Healthy and Heart Failure group presented from 40 to 91 years. The curves correspond to non-smoking females not taking medications.


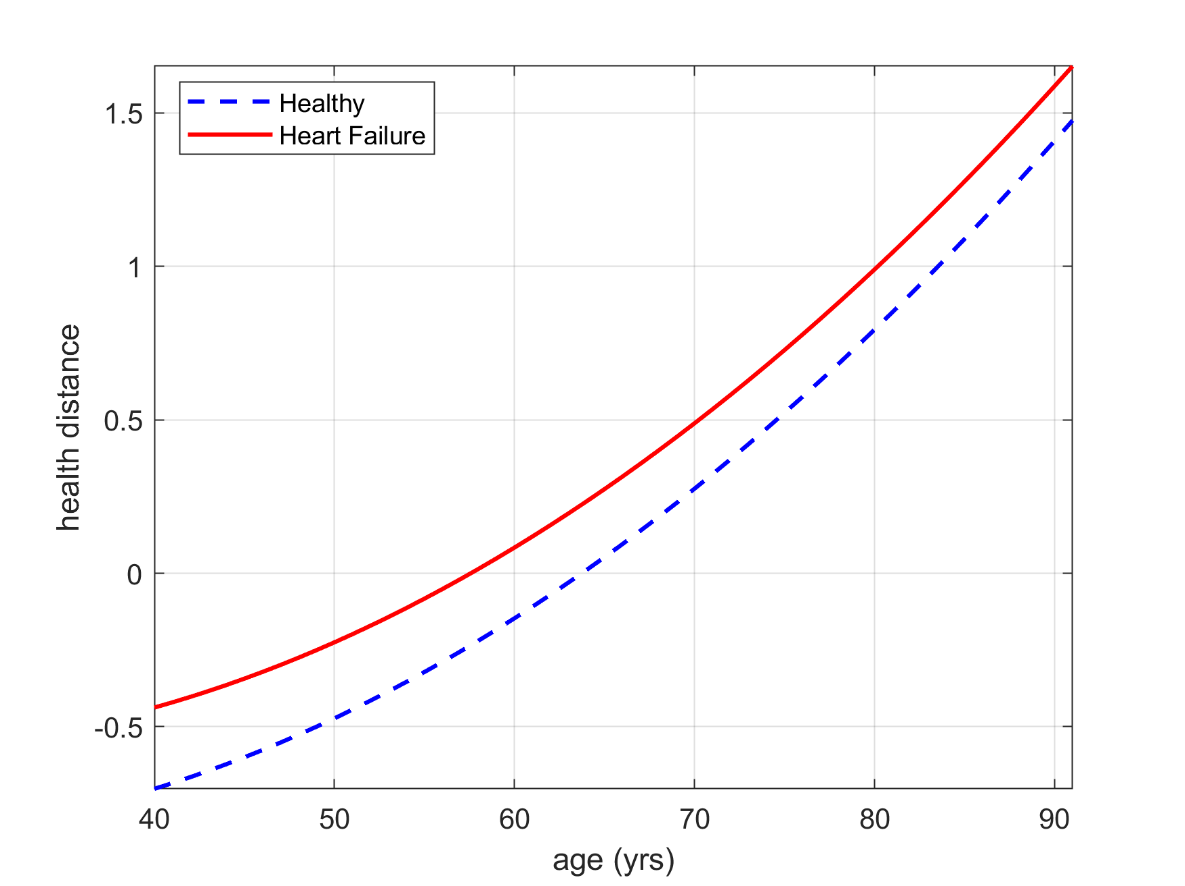


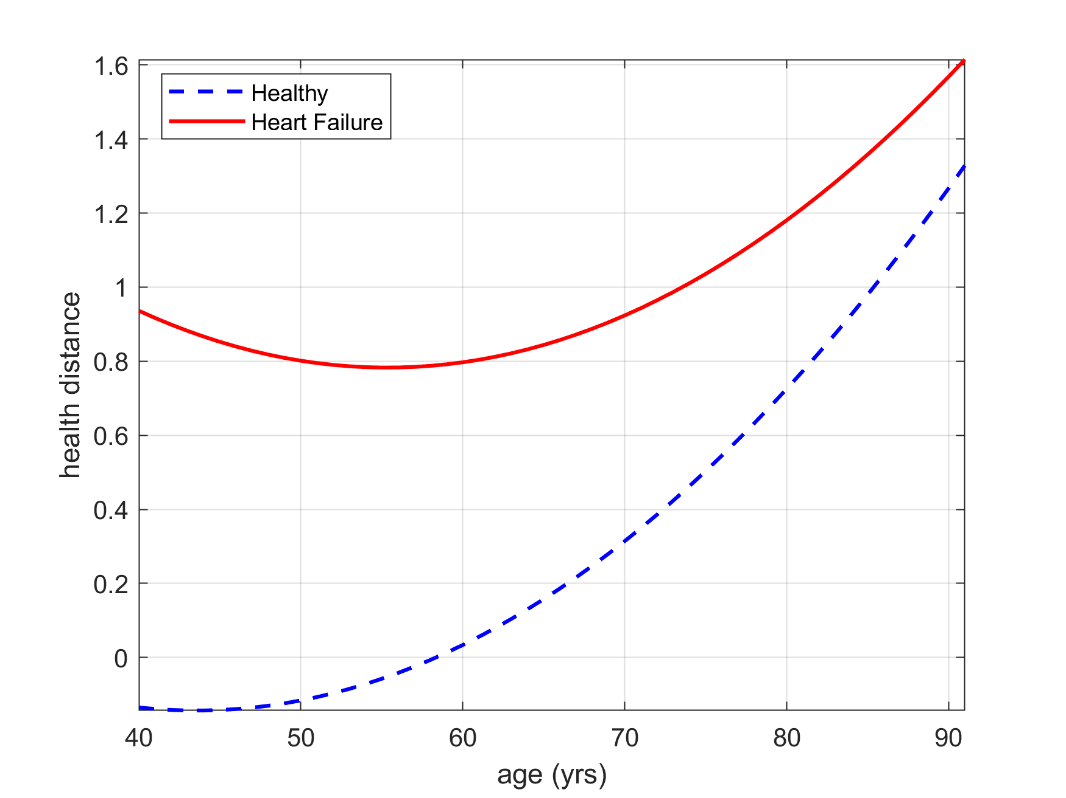
**Supplementary Figure S4.** Health distance trajectories for Neuromuscular coordination for the Healthy and Heart Failure group presented from 40 to 91 years. The curves correspond to non-smoking females not taking medications.

**Supplementary Figure S5.** Health distance trajectories for Physical activity for the Healthy and Heart Failure group presented from 40 to 91 years. The curves correspond to non-smoking females not taking medications.


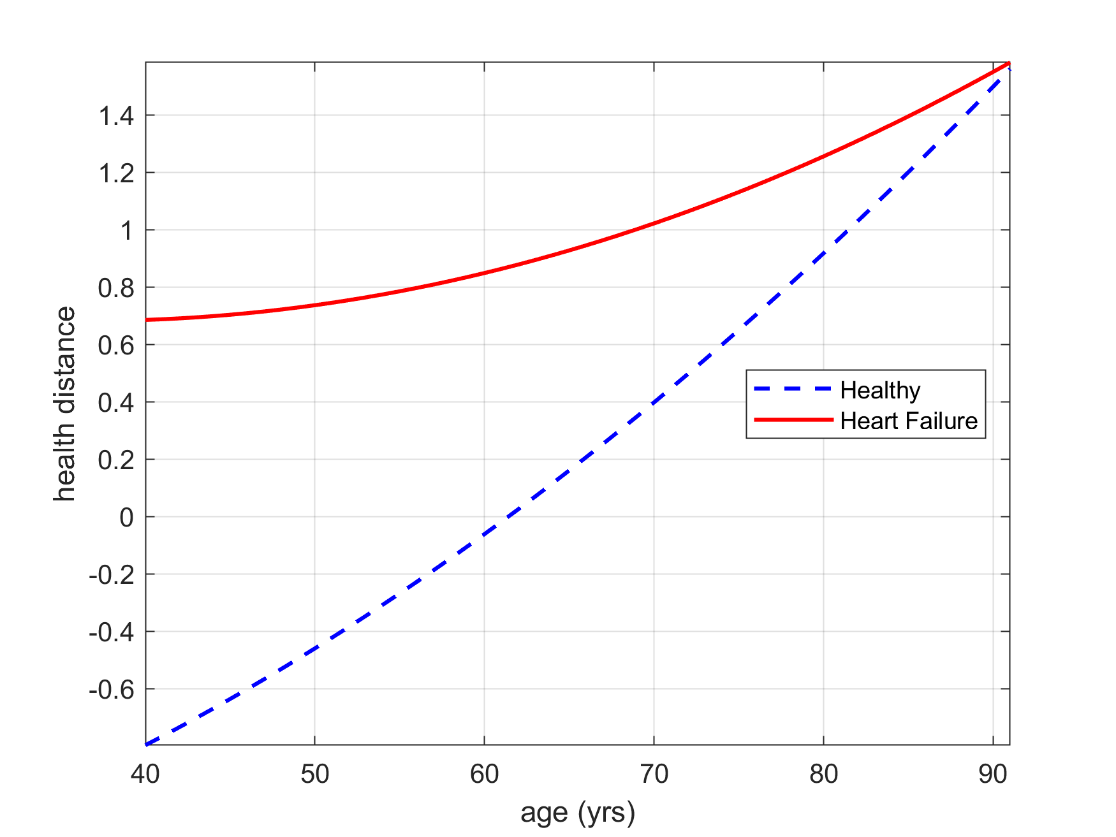


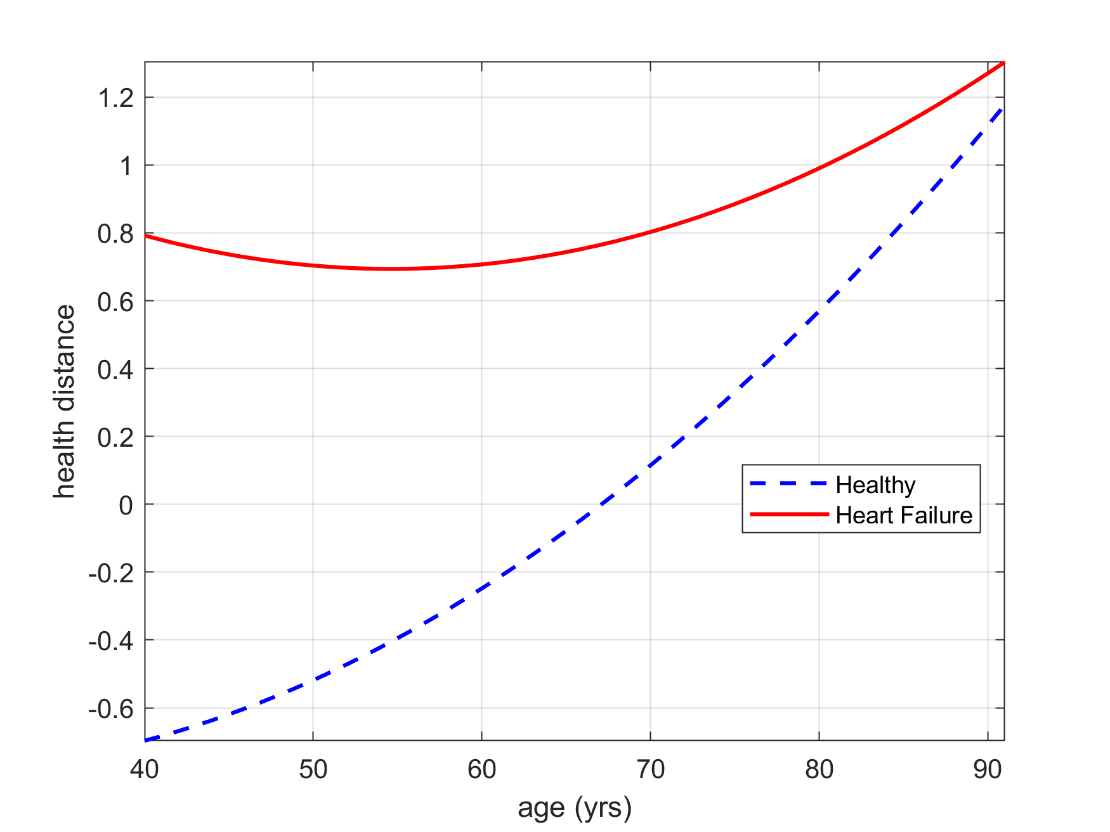
**Supplementary Figure S6.** Health distance trajectories for General health for the Healthy and Heart Failure group presented from 40 to 91 years. The curves correspond to non-smoking females not taking medications.

**Supplementary Figure S7.** Health distance trajectories for Anthropometry for the Healthy and Heart Failure group presented from 40 to 91 years. The curves correspond to non-smoking females not taking medications.


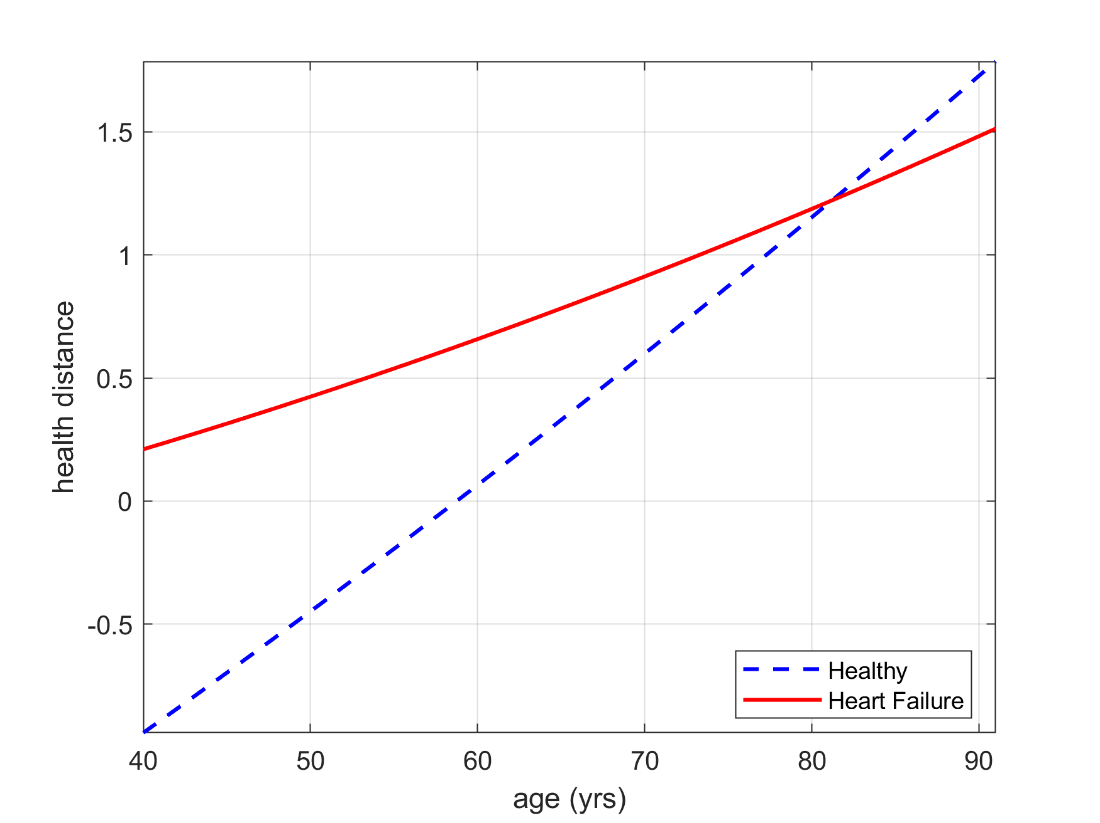

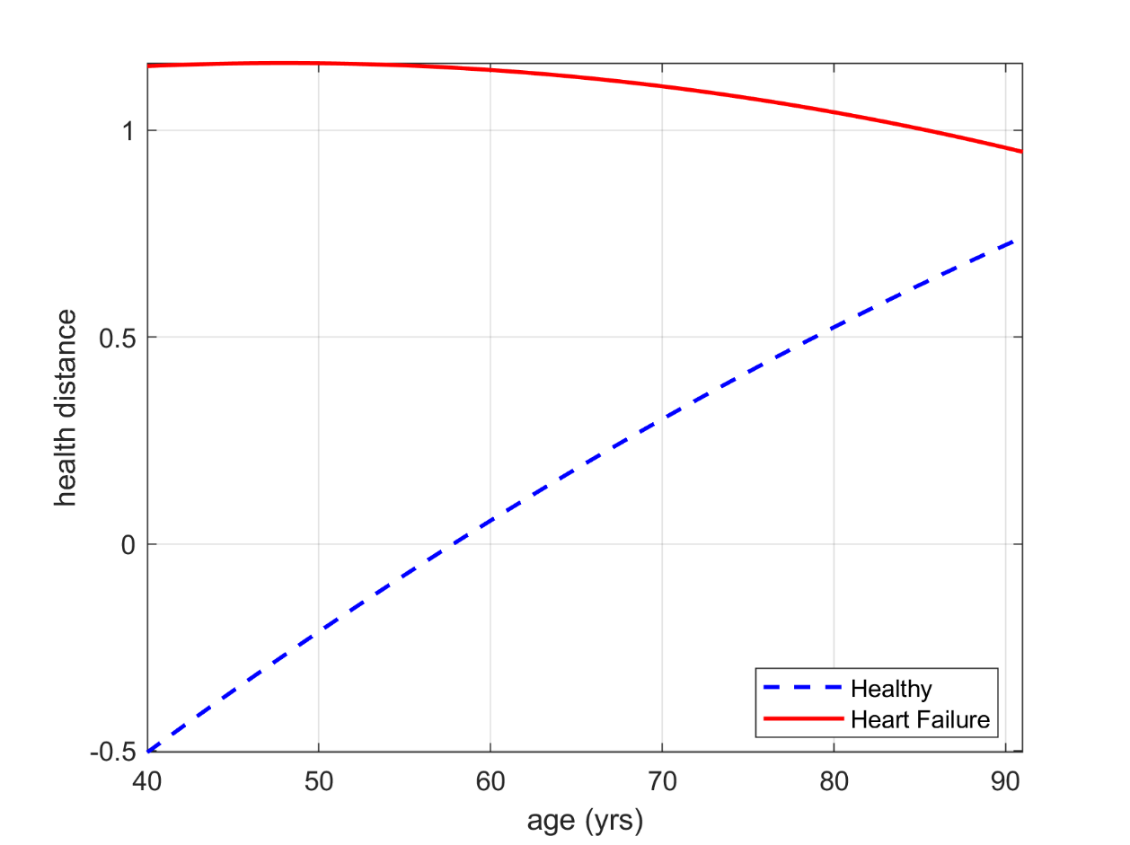
**Supplementary Figure S8.** Health distance trajectories for Blood biomarkers for the Healthy and Heart Failure group presented from 40 to 91 years. The curves correspond to non-smoking females not taking medications.

**Supplementary Figure S9.** Health distance trajectories for Vascular and respiratory health for the Healthy and Heart Failure group presented from 40 to 91 years. The curves correspond to non-smoking females not taking medications.

**
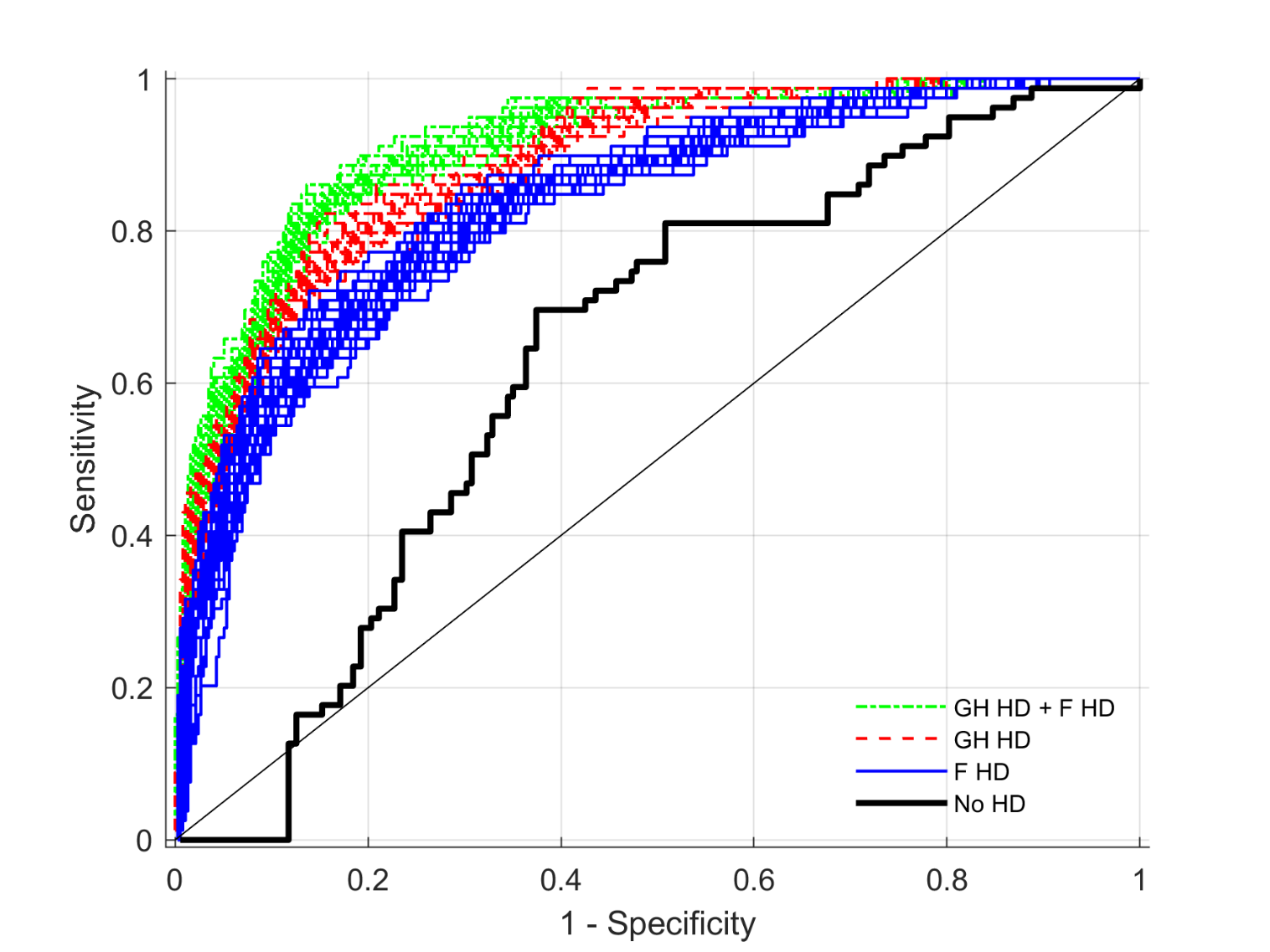
**

**Supplementary Figure S10.** Receiver operating characteristics (ROC) curves for Health distances (HD) of General health (GH), Fitness (F) and the combination of both HDs as predictors of Heart Failure. ROC curves presented for all imputed data sets.


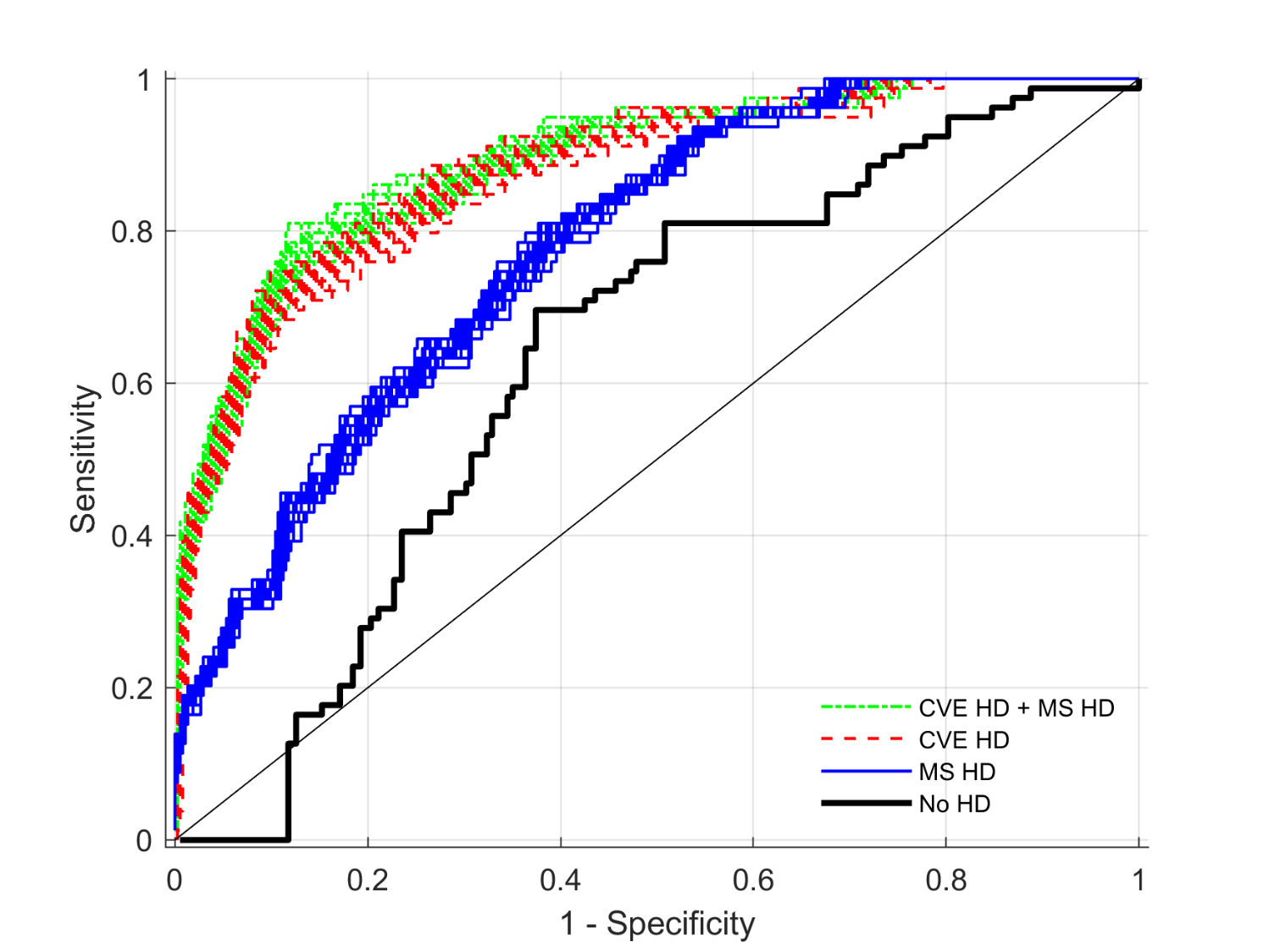


**Supplementary Figure S11.** Receiver operating characteristics (ROC) curves for Health distances (HD) of Cardiovascular endurance (CVE), Muscle strength (MS) and the combination of both HDs as predictors of Heart Failure. ROC curves presented for all imputed data sets.


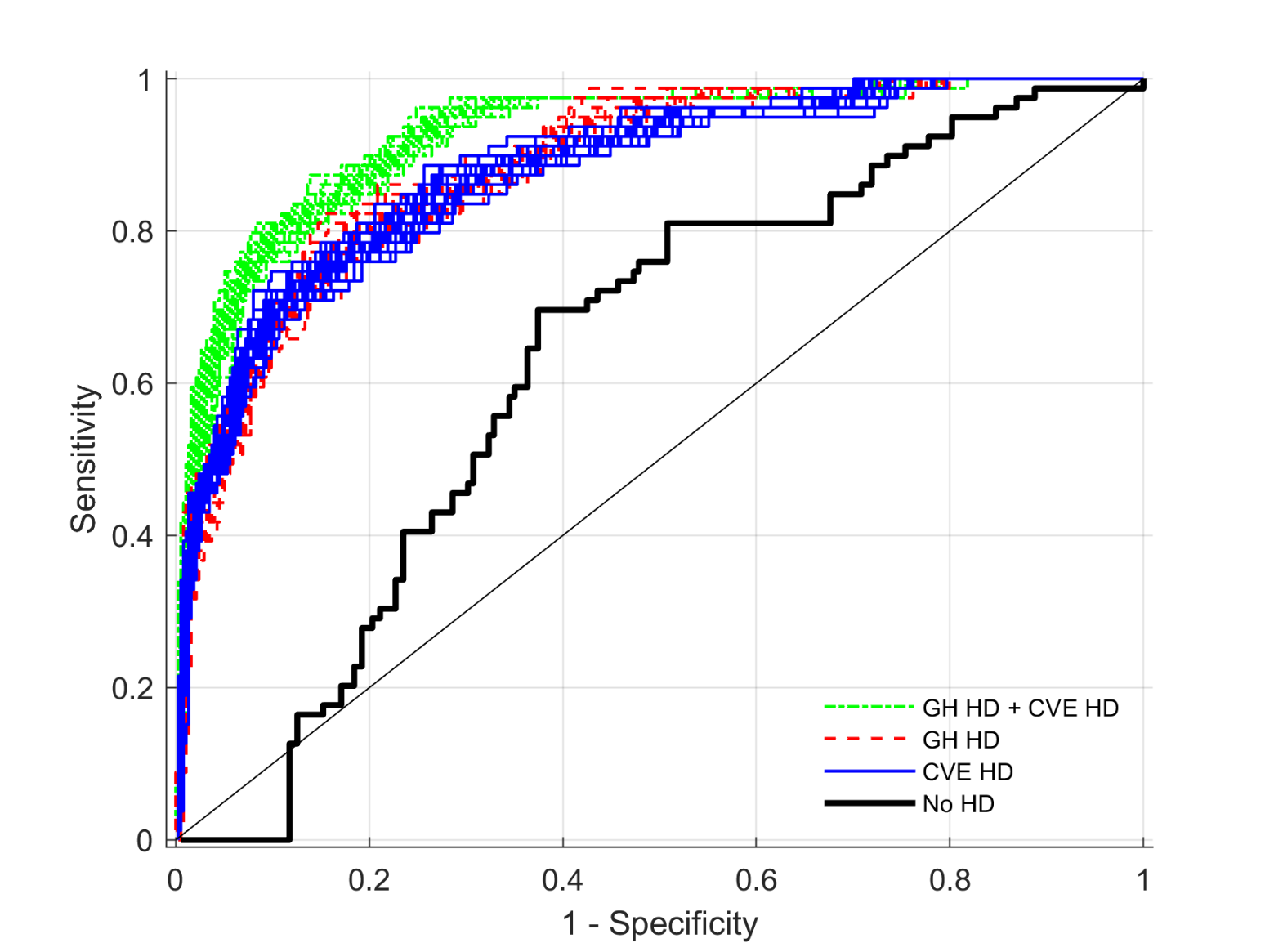
**Supplementary Figure S12.** Receiver operating characteristics (ROC) curves for Health distances (HD) of General health (GH), Cardiovascular endurance (CVE) and the combination of both HDs as predictors of Heart Failure. ROC curves presented for all imputed data sets.

## Supplementary Tables

| **Table S1.** Correlation of biomarkers with age. | | | |
| --- | --- | --- | --- |
| **Biomarker** | **Included in HD** | **Correlation coefficient** | ***p*-value** |
| **Anthropometry** |  |  |  |
| Height (cm) | 0 | -0.291 | < 0.0001 |
| Body mass (kg) | 1 | -0.014 | 0.7280 |
| BMI (kg/m2) | 0 | 0.197 | < 0.0001 |
| WHR | 1 | 0.430 | < 0.0001 |
| Body fat (%) | 1 | 0.449 | < 0.0001 |
| Lean body mass (kg) | 0 | -0.265 | < 0.0001 |
| Skeletal muscle mass (kg) | 1 | -0.303 | < 0.0001 |
| **Vascular and respiratory health** |  |  |  |
| Rest systolic BP (mmHg) | 0 | 0.412 | < 0.0001 |
| Rest diastolic BP (mmHg) | 0 | 0.339 | < 0.0001 |
| HR at rest (bpm) | 1 | 0.072 | 0.0786 |
| baPWV (m/s) | 1 | 0.760 | < 0.0001 |
| CAVI | 1 | 0.856 | < 0.0001 |
| Preejection period (ms) | 1 | 0.159 | < 0.0001 |
| Ejection time LV (ms) | 1 | 0.121 | 0.0028 |
| FVC | 1 | -0.582 | < 0.0001 |
| FEV1 | 0 | -0.647 | < 0.0001 |
| **Blood testing** |  |  |  |
| NTproBNP (pg/ml) | 0 | 0.257 | < 0.0001 |
| HbA1c (mg/dL) | 1 | 0.459 | < 0.0001 |
| Total cholesterol (mg/dL) | 1 | 0.298 | < 0.0001 |
| Triglyceride (mg/dL) | 1 | 0.059 | 0.1532 |
| HDL cholesterol (mg/dL) | 1 | 0.104 | 0.0108 |
| LDL cholesterol (mg/dL) | 0 | 0.275 | < 0.0001 |
| C-reactive protein (mg/L) | 1 | 0.100 | 0.0144 |
| Creatinine (mg/dl) | 1 | 0.154 | 0.0002 |
| **Cardiovascular endurance** |  |  |  |
| Peak V̇O_2_ (L/min) | 1 | -0.611 | < 0.0001 |
| Peak V̇O_2_ (mL/kg/min) | 1 | -0.671 | < 0.0001 |
| Peak V̇O_2_ (mL/kg leanmass/min) | 0 | -0.668 | < 0.0001 |
| Peak O_2_pulse (mL/beat) | 1 | -0.391 | < 0.0001 |
| Peak workload (W) | 0 | -0.628 | < 0.0001 |
| V̇O_2_ at VT1 (mL/kg/min) | 1 | -0.5369 | < 0.0001 |
| V̇O_2_ at VT1 (L/min) | 1 | -0.502 | < 0.0001 |
| PETCO_2_ at rest (mmHg) | 1 | -0.388 | < 0.0001 |
| PETCO_2_ at VT1 (mmHg) | 1 | -0.581 | < 0.0001 |
| V̇E/ V̇CO_2_ slope | 1 | 0.295 | < 0.0001 |
| V̇E/ V̇CO_2_ slope below VT2 | 1 | 0.509 | < 0.0001 |
| OUES (mL/min) | 1 | -0.525 | < 0.0001 |
| OUES (mL/min/kg) | 1 | -0.566 | < 0.0001 |
| % rel V̇O_2_ reduction 60sec post test | 1 | -0.426 | < 0.0001 |
| slope linear V̇O_2_ off-kinetics (ml/min/s) | 1 | 0.579 | < 0.0001 |
| Peak Lac (mmol/L) | 1 | -0.689 | < 0.0001 |
| Peak V̇E (l/min) | 0 | -0.551 | < 0.0001 |
| Peak HR (bpm) | 1 | -0.715 | < 0.0001 |
| HRR 1 min (bpm) | 1 | 0.159 | 0.0001 |
| HRR 2 min (bpm) | 1 | 0.403 | < 0.0001 |
| Peak exercise systolic BP (mmHg) | 1 | 0.064 | 0.1433 |
| **Muscle strength / power** |  |  |  |
| CMJ peak power (kN) | 1 | -0.600 | < 0.0001 |
| CMJ height (m) | 1 | -0.737 | < 0.0001 |
| Hand grip strength (N) | 1 | -0.383 | < 0.0001 |
| Hand grip RFD (N/150ms) | 1 | -0.453 | < 0.0001 |
| Isometric leg strength (kg) | 1 | -0.433 | < 0.0001 |
| **Neuromuscular coordination** |  |  |  |
| COP path length (cm) | 1 | 0.652 | < 0.0001 |
| Gait speed (m/s) | 1 | -0.234 | < 0.0001 |
| Gait cadence (steps/minute) | 1 | 0.136 | 0.0010 |
| Stride Length (m) | 1 | -0.395 | < 0.0001 |
| Gait double support (%) | 1 | -0.053 | 0.2089 |
| Gait asymmetry (%) | 1 | 0.147 | 0.0004 |
| **Physical activity** |  |  |  |
| Light physical activity (min/day) | 1 | 0.117 | 0.0050 |
| Moderate physical activity (min/day) | 1 | -0.352 | < 0.0001 |
| Vigorous physical activity (min/day) | 1 | -0.277 | < 0.0001 |

| **Table S2:** Group differences in biomarkers for Healthy (healthy participants aged ≥ 40 years) and Heart Failure (patients with heart failure). | | | | |
| --- | --- | --- | --- | --- |
| **Biomarker** | **Included in HD** | **Healthy (mean)** | **Reference Population (mean)** | ***p*-value** |
| **Anthropometry** |  |  |  |  |
| Height (cm) | 0 | 170.4 | 174.5 | < 0.0001 |
| Body mass (kg) | 1 | 69.8 | 70.2 | 0.6885 |
| BMI (kg/m2) | 0 | 23.9 | 23.0 | 0.0002 |
| WHR | 1 | 0.9 | 0.8 | < 0.0001 |
| Body fat (%) | 1 | 25 | 19 | < 0.0001 |
| Lean body mass (kg) | 0 | 52.3 | 56.7 | < 0.0001 |
| Skeletal muscle mass (kg) | 1 | 170.4 | 174.5 | < 0.0001 |
| **Vascular and respiratory health** |  |  |  |  |
| Rest systolic BP (mmHg) | 0 | 28.8 | 31.9 | < 0.0001 |
| Rest diastolic BP (mmHg) | 0 | 130 | 121 | < 0.0001 |
| HR at rest (bpm) | 1 | 79 | 72 | 0.8524 |
| baPWV (m/s) | 1 | 61 | 61 | < 0.0001 |
| CAVI | 1 | 13.3 | 10.2 | < 0.0001 |
| Preejection period (ms) | 1 | 8.8 | 6.2 | < 0.0001 |
| Ejection time LV (ms) | 1 | 105.8 | 98.9 | 0.0017 |
| FVC | 1 | 313.1 | 307.6 | < 0.0001 |
| FEV1 | 0 | 3.9 | 5.0 | < 0.0001 |
| **Blood testing** |  |  |  |  |
| NTproBNP (pg/ml) | 0 | 138.0 | 79.2 | < 0.0001 |
| HbA1c (mg/dL) | 1 | 5.3 | 5.0 | < 0.0001 |
| Total cholesterol (mg/dL) | 1 | 232 | 191 | < 0.0001 |
| Triglyceride (mg/dL) | 1 | 119 | 113 | 0.2969 |
| HDL cholesterol (mg/dL) | 1 | 67.1 | 62.0 | 0.0006 |
| LDL cholesterol (mg/dL) | 0 | 130.6 | 102.6 | < 0.0001 |
| C-reactive protein (mg/L) | 1 | 1.93 | 1.60 | 0.3875 |
| Creatinine (mg/dl) | 1 | 0.84 | 0.83 | 0.2574 |
| **Cardiovascular endurance** |  |  |  |  |
| Peak V̇O_2_ (L/min) | 1 | 2.22 | 3.00 | < 0.0001 |
| Peak V̇O_2_ (mL/kg/min) | 1 | 31.7 | 42.9 | < 0.0001 |
| Peak V̇O_2_ (mL/kg leanmass/min) | 0 | 41.8 | 52.9 | < 0.0001 |
| Peak O_2_pulse (mL/beat) | 1 | 14.1 | 16.6 | < 0.0001 |
| Peak workload (W) | 0 | 182 | 257 | < 0.0001 |
| V̇O_2_ at VT1 (mL/kg/min) | 1 | 19.5 | 24.8 | < 0.0001 |
| V̇O_2_ at VT1 (L/min) | 1 | 1.4 | 1.7 | < 0.0001 |
| PETCO_2_ at rest (mmHg) | 1 | 31.0 | 32.6 | < 0.0001 |
| PETCO_2_ at VT1 (mmHg) | 1 | 39.7 | 43.7 | < 0.0001 |
| V̇E/ V̇CO_2_ slope | 1 | 37.3 | 34.5 | < 0.0001 |
| V̇E/ V̇CO_2_ slope below VT2 | 1 | 30.5 | 26.8 | < 0.0001 |
| OUES (mL/min) | 1 | 2376 | 3031 | < 0.0001 |
| OUES (mL/min/kg) | 1 | 33.9 | 43.3 | < 0.0001 |
| % rel V̇O_2_ reduction 60sec post test | 1 | 28.3 | 33.9 | < 0.0001 |
| slope linear V̇O_2_ off-kinetics (ml/min/s) | 1 | -13.4 | -19.9 | < 0.0001 |
| Peak Lac (mmol/L) | 1 | 7.0 | 10.3 | < 0.0001 |
| Peak V̇E (l/min) | 0 | 98 | 128 | < 0.0001 |
| Peak HR (bpm) | 1 | 162 | 188 | < 0.0001 |
| HRR 1 min (bpm) | 1 | -24 | -25 | 0.8200 |
| HRR 2 min (bpm) | 1 | -52 | -59 | < 0.0001 |
| Peak exercise systolic BP (mmHg) | 1 | 190.7 | 181.3 | < 0.0001 |
| **Muscle strength / power** |  |  |  |  |
| CMJ peak power (kN) | 1 | 2.1 | 2.9 | < 0.0001 |
| CMJ height (m) | 1 | 0.16 | 0.26 | < 0.0001 |
| Hand grip strength (N) | 1 | 344.0 | 412.7 | < 0.0001 |
| Hand grip RFD (N/150ms) | 1 | 226.8 | 280.9 | < 0.0001 |
| Isometric leg strength (kg) | 1 | 104 | 135 | < 0.0001 |
| **Neuromuscular coordination** |  |  |  |  |
| COP path length (cm) | 1 | 42.0 | 23.9 | < 0.0001 |
| Gait speed (m/s) | 1 | 1.4 | 1.4 | 0.1855 |
| Gait cadence (steps/minute) | 1 | 115 | 113 | 0.0034 |
| Stride Length (m) | 1 | 1.46 | 1.50 | < 0.0001 |
| Gait double support (%) | 1 | 21.5 | 21.9 | 0.1846 |
| Gait asymmetry (%) | 1 | 2.6 | 2.2 | 0.0476 |
| **Physical activity** |  |  |  |  |
| Light physical activity (min/day) | 1 | 103 | 94 | 0.0005 |
| Moderate physical activity (min/day) | 1 | 157 | 178 | < 0.0001 |
| Vigorous physical activity (min/day) | 1 | 6 | 9 | 0.0041 |

**Table S3.** Correlation of biomarkers.

Please see separate Excel file.
